# Identification and characterization of two conserved G-quadruplex forming motifs in the Nipah virus genome and their interaction with G-quadruplex specific ligands

**DOI:** 10.1038/s41598-020-58406-8

**Published:** 2020-01-30

**Authors:** Prativa Majee, Subodh Kumar Mishra, Nirali Pandya, Uma Shankar, Sanjeev Pasadi, K. Muniyappa, Debasis Nayak, Amit Kumar

**Affiliations:** 1 Discipline of Biosciences and Biomedical Engineering, Indian Institute of Technology Indore, Simrol, Indore, 453552 India; 2 Department of Biochemistry, Indian Institute of Science, Bangalore, 560012 India

**Keywords:** DNA, Genome informatics, Viral infection

## Abstract

The G-quadruplex (GQ) motifs are considered as potential drug-target sites for several human pathogenic viruses such as Zika, Hepatitis, Ebola, and Human Herpesviruses. The recent outbreaks of Nipah virus (NiV) in India, the highly fatal emerging zoonotic virus is a potential threat to global health security as no anti-viral drug or vaccine in currently available. Therefore, here in the present study, we sought to assess the ability of the putative G-quadruplex forming sequences in the NiV genome to form G-quadruplex structures and act as targets for anti-viral compounds. Bioinformatics analysis underpinned by various biophysical and biochemical techniques (such as NMR, CD, EMSA, DMS footprinting assay) confirmed the presence of two highly conserved G-quadruplex forming sequences (HGQs) in the G and L genes of NiV. These genes encode the cell attachment glycoprotein and RNA-dependent RNA polymerase, respectively and are essential for the virus entry and replication within the host cell. It remains possible that stabilization of these HGQs by the known G-quadruplex binding ligands like TMPyP4 and Braco-19 represents a promising strategy to inhibit the expression of the HGQ harboring genes and thereby stop the viral entry and replication inside the host cell. Accordingly, we report for the first time, that HGQs in Nipah virus genome are targets for G-quadruplex specific ligands; therefore, could serve as potential targets for anti-viral therapy.

## Introduction

The recent outbreaks of Nipah virus in India during May-June 2018 have caused much concern among the public because of its ease of transmission. NiV is a deadly zoonotic virus that can be transmitted from animals to humans. The virus belongs to the *Henipavirus* genus of the *Paramyxoviridae* family. By the severity and degree of the pathogenesis, NiV is classified as a biosafety level 4 pathogen and designated as category C priority pathogen^1^. The fruit bats of the *Pteropus* genus are believed to be the natural reservoir of this virus, thus helping in the spread of the virus among other animals including pigs and humans^2^. The viral infection can range from being asymptomatic to certain acute respiratory complications. In severe conditions, it even causes fatal encephalitis leading to a high fatality rate ranging from 40–75%. First reported in Kampung Sungai Nipah of Malaysia in 1998, from where it derives its name, this virus is prevalent only in certain regions of the world especially in South-East Asian countries. However, the repercussion of this disease results in high morbidity and mortality^3^. According to the Directorate of Health Services, Kerala, India, the recent NiV outbreaks exhibited a very high mortality rate of 88.9%^4^. Both animal-to-human and human-to-human transmissions are noted in the case of Nipah viral infections. Unavailability of licensed vaccines or drugs against this virus exacerbates the situation and begets the need for efficient drug targets^5^.

**Figure 1:**
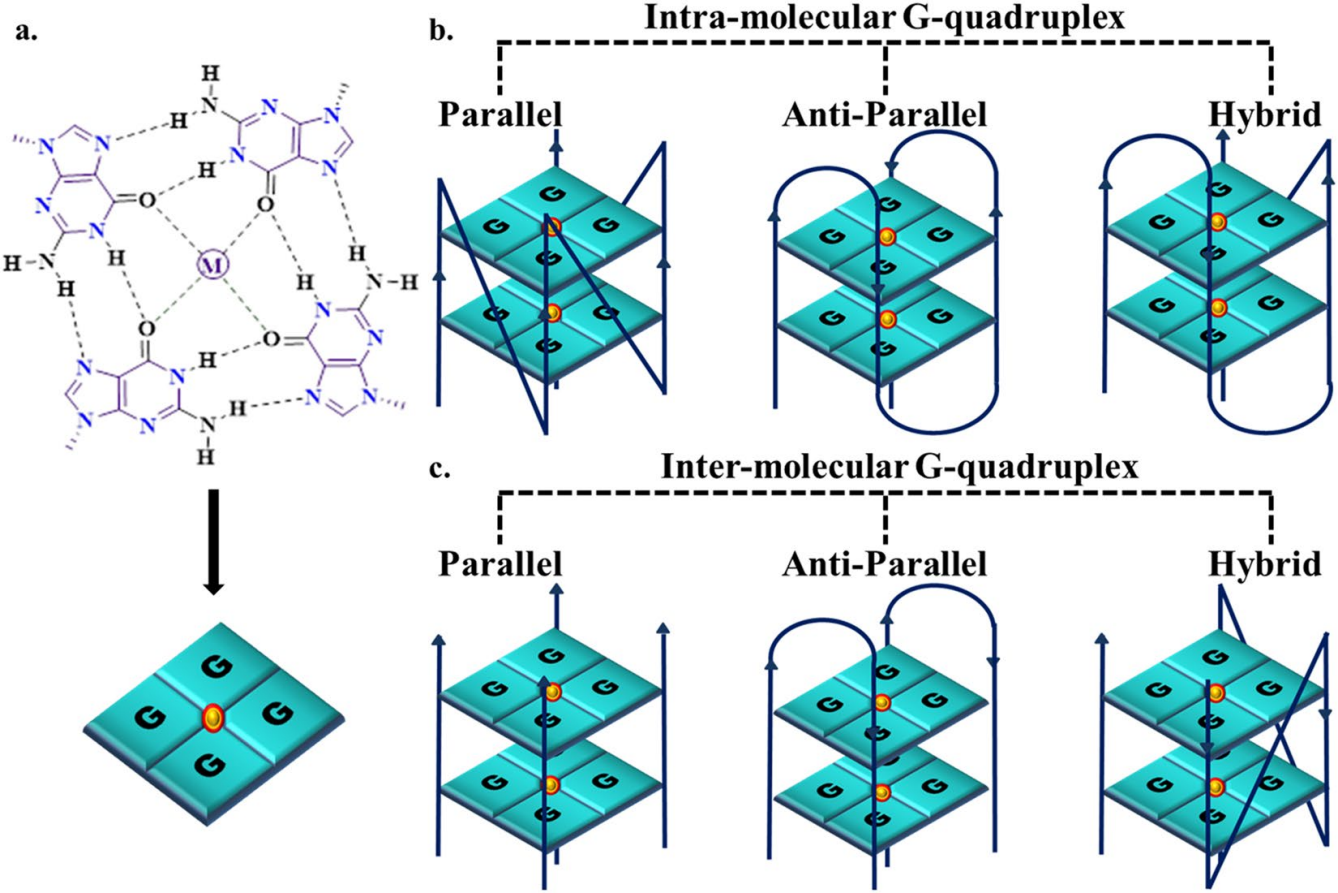
**(a)** The G-quartet structure formed by Hoogsteen base pairing of four guanine residues. (**b)** The intra-molecular topology of the G-quadruplex structure forming parallel, anti-parallel and hybrid conformations. **(c)** The inter-molecular topology of the G-quadruplex structure forming parallel, anti-parallel and hybrid conformations.

Numerous studies on G-quadruplexes have opened up avenues for the development of effective anti-viral therapeutics. G-quadruplexes are non-B secondary structures formed by guanine-rich sequences in DNA or RNA. The guanine residues associate with each other to form a quartet mediated by Hoogsteen hydrogen bonds and these quartets stack on top of each other to form the complete structure (Fig. 1). They are stabilized by cations which neutralize the negative charges of the carbonyl groups in the guanine residues. The stability and topology of these structures is primarily governed by the length and number of individual G-tracts, loop length, loop composition and  physiological conditions^6,7^. The GQ structures possess specific pockets that allow small molecules to stack or intercalate, thereby providing higher stability to these structures. G-quadruplex structures are viewed as potential drug targets in cancer biology, because of their ability to attenuate the telomerase activity^8^. A potent drug called Quarfloxin, which stabilizes the G-quadruplex in the telomeric region, has reached phase II clinical trials^9^. In addition to the telomere, the promoter regions of different genes like the human insulin gene, c-Myc gene, c-Kit gene, bcl2 gene, are reported to be enriched with the G-quadruplex structures asserting their role as transcriptional regulators^10^. The UTR of some genes is also enriched with G-quadruplex structures suggesting their involvement in transcription and translation^11^. Several studies have also confirmed the partaking of these structures in certain neurodegenerative diseases^12^. G-quadruplex sequences are also evolutionary conserved across different living organisms. They are present in essential genomic loci of *Saccharomyces cerevisiae*^13,14^*, Plasmodium falciparum*^15,16^*, Neiserria gonorrhea*^17^, *Mycobacterium tuberculosis*^18^, *Streptococcus pneumononiae*^19^ and several viruses^20^. They are also found in the genomes of plants including *Arabidopsis thaliana*, and *Oryza sativa*^21,22^. A recent review on G-quadruplexes in viruses and ligands targeting them emphasize the potential of G-quadruplex structures as novel anti-viral targets^20^. Genome-wide screening in Human Papillomavirus, Zika virus, Human Herpes Simplex Virus, etc. indicate that conserved G-rich sequences are capable of forming G-quadruplex structures^23–26^. Some of the known efficient G-quadruplex binding ligands namely TMPyP4, Braco-19, PDS, etc. are shown to stabilize conserved G-quadruplex structures present in viruses such as hepatitis C virus (HCV), Ebola virus, etc. which results in reduced gene expression^27,28^. Interestingly, a potential G-quadruplex in the promoter region of preS2/S gene of Hepatitis B virus results in up-regulated gene expression^29^. Collectively, these reports deduced the role of G-quadruplex structures in viral gene modulation and have propelled researchers to explore more into the role of G-quadruplexes in the viral life cycle.

Given the importance of G-quadruplex structures in viral life cycle, we sought to explore the presence of conserved G-quadruplex forming sequences (HGQs) in the NiV genome. There is significant sequence variability among different isolates of NiV, thus targeting the most conserved sequences could be efficacious. The genome-wide screening revealed two HGQs those are conserved in all the available twelve complete genomes of Nipah virus. The Nipah virus genome consists of six gene-encoded proteins namely nucleoprotein (N gene), phosphoprotein (P gene), matrix protein (M gene), fusion protein (F gene), glycoprotein (G gene) and large RNA dependent RNA polymerase (L gene). While the L gene is absolutely essential for negative-stranded RNA genome containing NiV replication as the host cell lacks this enzyme, the glycoprotein aids in the viral entry by recognizing and anchoring to the host ephrin-B2 and ephrin-B3 receptors. Thus the two conserved HGQs, namely, HGQ-NV-L and HGQ-NV-G present in the L gene and G gene respectively may have potential significance. Interestingly, the two known Nipah virus clades i.e. the Malaysian and the Bangladeshi clades of NiV contain certain other HGQs, which are conserved, in one of the two clades. The bioinformatics analysis supported by the biophysical assays including the CD spectra analysis, ^1^H NMR analysis, EMSA and DMS footprinting assay confirm the formation of stable G-quadruplex structures by the HGQs *in vitro*.

Biophysical studies with the known G-quadruplex binding ligands, TMPyP4 and Braco-19, also showed that Nipah HGQ sequences exhibit strong affinity for these molecules. Furthermore, we found Taq DNA polymerase arrest due to formation of stable G-quadruplex structures. The HGQs are present in the ORF region of the two genes and stabilization of these structures may result in the suppression of the gene expression which was validated by the mTFP reporter assay. Earlier studies have also showed that the stable G-quadruplex sequences in the ORF regions may lead to stalling of the transcriptional or translational machinery resulting in suppression of gene expression^30,31^. Taken together, this study supports the existence of G-quadruplex forming sequences in the NiV genome and that these structures could serve as targets for anti-viral therapy.

## Results

### Bioinformatics analysis reveals putative G-quadruplex forming sequences in NiV genome

The nucleotide sequence comprising of at least two or more consecutive guanine residues separated by a spacer region forms G-quadruplex structures. However, only two G-tetrads are enough to form the G-quadruplex structures as depicted in viruses such as Florida manatee species infecting papillomavirus and Pseudorabies virus^32,33^. Following this notion, we performed the genome-wide screening of potential G-quadruplex forming sequences in all available isolates of NiV (Table S1) by using our previously published G-quadruplex prediction tool^34^ (Table S2). The predictions were further confirmed by the other online tools like QGRS mapper^35^ and QuadBase2^36^ (Tables S3 and S4). Evolutionarily conserved sequence motifs in an organism signify their importance in the survival of the organism. Therefore, the frequency of conservation of these HGQs among the different isolates of the virus was predicted and enlisted in Table S11.

**Figure 2:**
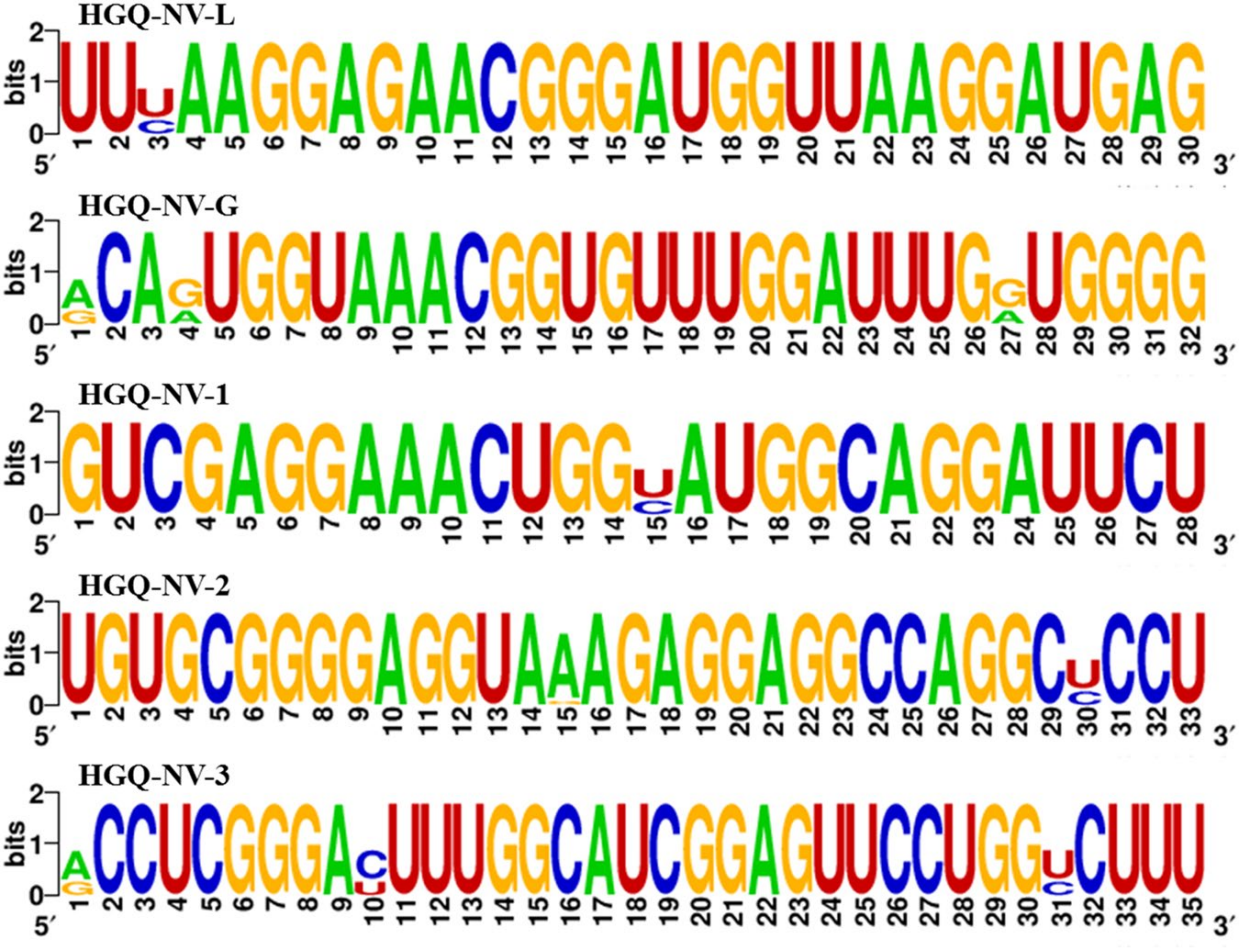
Sequence logos constructed for the five conserved HGQs predicted in the NiV genome using the WebLogo software^59^. While highly conserved nucleotides are represented by a larger font-size, the largely variable nucleotides are represented by smaller font-size. All other sequence logo constructs are shown in Fig. S7.

The genome-wide mining revealed the presence of five potential, highly conserved G-quadruplex structure forming motifs (HGQs), which are conserved among all the twelve viral isolates (Fig. 2; Table S8). Additionally, the complete genome sequences of all the twelve viral isolates were aligned and the sequence logos of all the predicted G-quadruplex sequences were further generated (Figs. S6 and S7). Our findings for Nipah virus are in compliance with a recent study encompassing a database regarding all the putative G-quadruplex sequences in human infecting viruses (Fig. S5)^37^.

Further, the NiV strains are classified into two clades: Malaysia clade enclosing eight isolates and Bangladesh clade which includes four isolates and fascinatingly certain G-quadruplex forming sequences were found to be conserved exclusively in one of the two clades (Tables S9 and S10). The Bangladeshi clade has diverged from the Malaysian clade to be more pathogenic^38^ and whether these specific HGQs possess any significance on the evolution and proliferation of the virus of the particular clade will be an intriguing aspect to be explored.

Interestingly, when we analyzed for the genomic location of the HGQs, we found that all of the G-quadruplex sequences are present in the protein-coding region of the essential viral genes (Table S11). Especially, the HGQ-NV-G and HGQ-NV-L are present in the coding region of the glycoprotein G gene and the RNA dependent RNA polymerase, L gene respectively which are extremely essential for the virus entry and replication. Nipah virus being a (−ve) stranded RNA virus, transcribes the (+)ve sense transcripts i.e. the mRNA from its (−)ve sense genome before the subsequent translation of viral proteins. Additionally, during viral genome replication, the complementary (+)ve strand serves as the template for subsequent synthesis of genomic (−)ve strand RNA. Thus, HGQ search in both the strands proves to be beneficial as both of them are equally involved in virus biology.

### NMR spectroscopic analysis confirms the presence of Hoogsteen hydrogen bonds in the G-quadruplex structures

**Figure 3:**
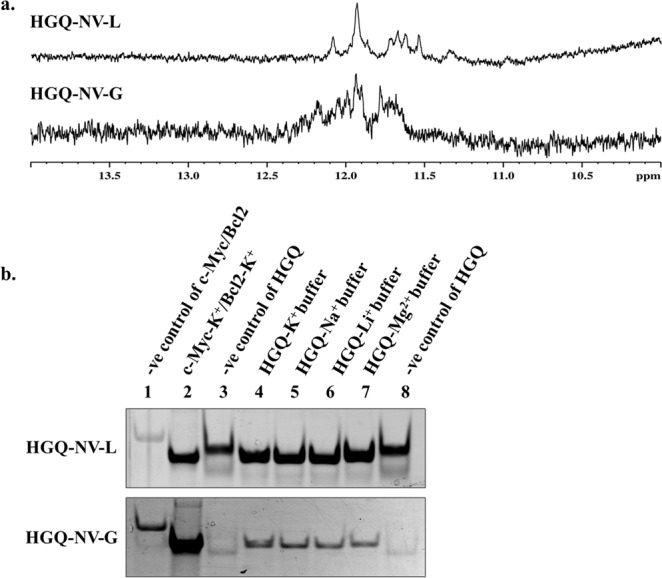
(**a**) NMR spectra analysis. 1D ^1^H NMR exhibiting the chemical shift between 10–12 ppm arising due to the presence of imino-proton groups in both the HGQs. (**b**) Electrophoretic Gel Mobility Shift Assay. The mobility shifts of the HGQ sequences in presence of cations shown as compared to their mutant sequences. Lane 3 and 8 denote the mobility of the linear mutant counterparts of the HGQs, Lane 4, 5, 6 and 7 denote the mobility of HGQs in the presence of K^+^, Na^+^, Li^+^, and Mg^2+^ respectively. Lane 2 represents the mobility of the known G-quadruplex forming sequence, c-Myc (was used in case of HGQ-NV-L) and bcl2 (was used in case of HGQ-NV-G) acting as the positive control while lane 1 is the negative control of c-Myc or bcl2, i.e., the mutant sequence of c-Myc or bcl2 gene. Full-length gels are shown in Fig. S19.

Since bioinformatics analysis for G-quadruplex prediction is not adequate to confirm the formation of G-quadruplex structures, we carried out biophysical experiments with the predicted HGQs. Nuclear Magnetic Resonance (NMR) spectroscopy has proven to be an essential experimental tool for G-quadruplex studies. While high-resolution solution structures can be determined by NMR spectroscopy, the basic 1D ^1^H NMR gives us an idea about the guanine imino-protons involved in G-quadruplex formation. The Hoogsteen base pairing in G-quadruplex structures reveals a chemical shift in the 10–12 ppm range which is distinctively different from the Watson-Crick base pairing exhibiting a chemical shift in the 13–14 ppm region^39^. Among the five HGQs, which are conserved in all the virus isolates, only two HGQs namely, HGQ-NV-L and HGQ-NV-G formed G-quadruplex structures *in vitro* (Fig. S12). Both the HGQ-NV-L and HGQ-NV-G of the NiV were seen to exhibit a chemical shift in the imino-proton region of 10–12 ppm confirming the formation of Hoogsteen base pairing in the G-quadruplex sequences (Fig. 3a). Therefore, all the further studies were performed with these two HGQs.

### Molecularity of G-quadruplex structures

EMSA is widely used as an experimental tool to assess the molecularity of G-quadruplex structures. The intra-molecular G-quadruplex structures form a compact topology and run faster in the gel as compared to their linear counterparts. In the case of inter-molecular G-quadruplex structure, association of more than one nucleic acid strand in G-quadruplex formation results in slower mobility (due to higher molecular weight) of the complex compared to their linear equivalent thus allowing the HGQ band to run slower^22,40^. The HGQ-NV-L sequence was seen to migrate faster in comparison to its linear mutant, thereby suggesting the formation of intra-molecular G-quadruplex structure. On the other hand, the HGQ-NV-G was seen to run slower relative to its mutant, indicating the formation of inter-molecular G-quadruplex conformation (Fig. 3b).

The HGQ sequences were also evaluated by the dimethyl sulfate (DMS) footprinting assay which ascertained the formation of GQ structures. In the presence of K^+^ ion, the guanines of the HGQ-NV-G sequence involved in the inter-molecular GQ structure formation are protected, therefore the residues corresponding to G23-G29 stretch were not cleaved (Fig. S13b). This is because these guanine residues are paired by the Hoogsteen hydrogen bonds which restrict the methylation of these residues. Nevertheless, in the case of HGQ-NV-L (Fig. S13a), the bands corresponding to the guanines involved in the GQ formation have not completely disappeared but there is significant change in intensity as compared to the single stranded DNA (no K^+^ added) which attests that the protection is due to GQ formation.

### Effect of different ions on topology of the G-quadruplex structures

**Figure 4:**
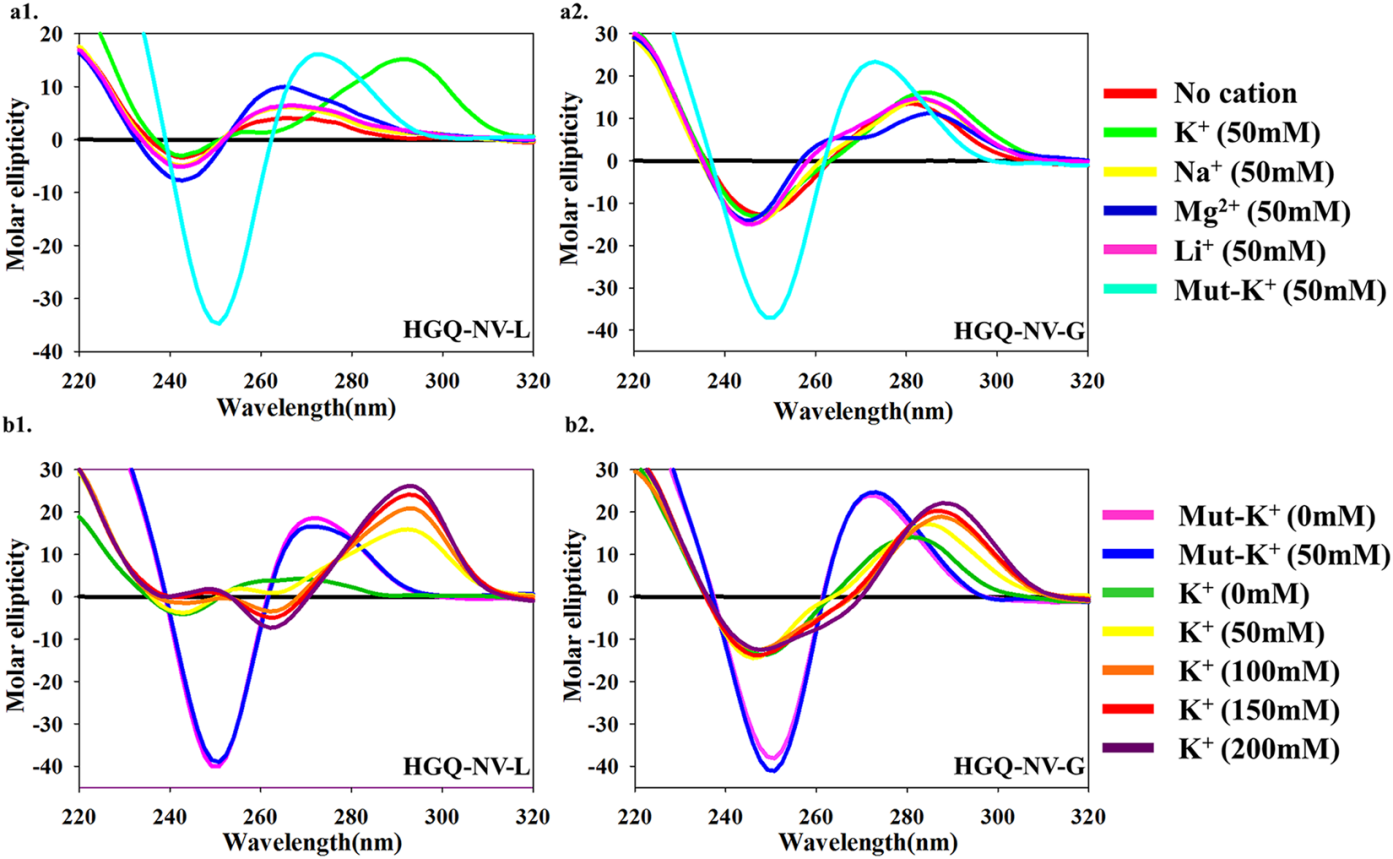
(**a**) CD spectra analysis in the presence of different cations. CD spectra scan of both the HGQs in Tris-HCl buffer containing K^+^, Na^+^, Mg^2+^or Li^+^ along with their respective mutants (a1: HGQ-NV-L; a2: HGQ-NV-G). (**b**) CD spectra analysis with an increasing concentration of potassium. The CD spectra scan for both the HGQs with increasing concentration of K^+^ ion along with their respective mutants (b1: HGQ-NV-L; b2: HGQ-NV-G).

CD spectroscopy is a valuable biophysical tool for determining the folding topology of non-canonical G-quadruplex structures. The parallel G-quadruplex topology exhibits a positive peak at ~265 nm and a negative peak at ~240 nm while the anti-parallel G-quadruplex topology exhibits a positive peak at ~290 nm followed by a negative peak at ~260 nm. On the other hand, hybrid G-quadruplex topology displays a CD spectra with a positive peak both at ~265 nm and ~290 nm along with a negative peak at ~240 nm^41^. The cations play an important role in stabilizing the G-quadruplex structure by nullifying the negatively charged center of the quartets. Thus the CD spectra of both the HGQs were observed in four different cationic conditions including potassium, sodium, lithium, and magnesium. K^+^, Na^+^ and Mg^2+^ are physiologically relevant cations while Li^+^ is used in control experiments as it does not show much effect on G-quadruplex stability^42^. HGQ-NV-G was found to exhibit hybrid topology in the presence of all these cations (Fig. 4a2). Whereas HGQ-NV-L showed to bear hybrid topology in the presence of K^+^ ion but parallel topology in the presence of the other three cations (Fig. 4a1). As expected the mutant sequences of all the HGQs displayed a deep negative peak at ~250 nm and a positive peak at ~270 nm that is the characteristic pattern of non-G-quadruplex B-form structure (Fig. 4a).

Potassium ion is known to stabilize the G-quadruplex structures better because of its atomic size and ionic radii^43,44^. Accordingly, we observed a higher CD ellipticity in the presence of K^+^ for both the HGQs. Few studies posit that the viral infection results in the change in host intracellular ion concentration specifically in K^+^ concentration^45^. Therefore, we explored the stability of the putative G-quadruplex in the presence of K^+^ ion, lower as well as higher concentrations than the cytosolic concentration i.e. ~100 mM. In the absence of K^+^ ion (K^+^  = 0 mM), both the HGQs did not display the typical CD spectra for G-quadruplex, but with the gradual increase in K^+^ concentration, from 50 mM to 200 mM, the ellipticity of the CD spectra was observed to increase markedly in both the HGQs (Fig. 4b1,b2). The increasing ellipticity with increasing K^+^ concentration depicts the higher stability of these secondary structures. Interestingly, a significant transition was seen from hybrid topology to anti-parallel topology in case of HGQ-NV-L with increase in the K^+^ ion concentration from 50 mM to 100 mM (Fig. 4b1).

### Binding of G-quadruplex specific ligands to the Nipah HGQs analyzed through Isothermal Calorimetry (ITC)

**Figure 5:**
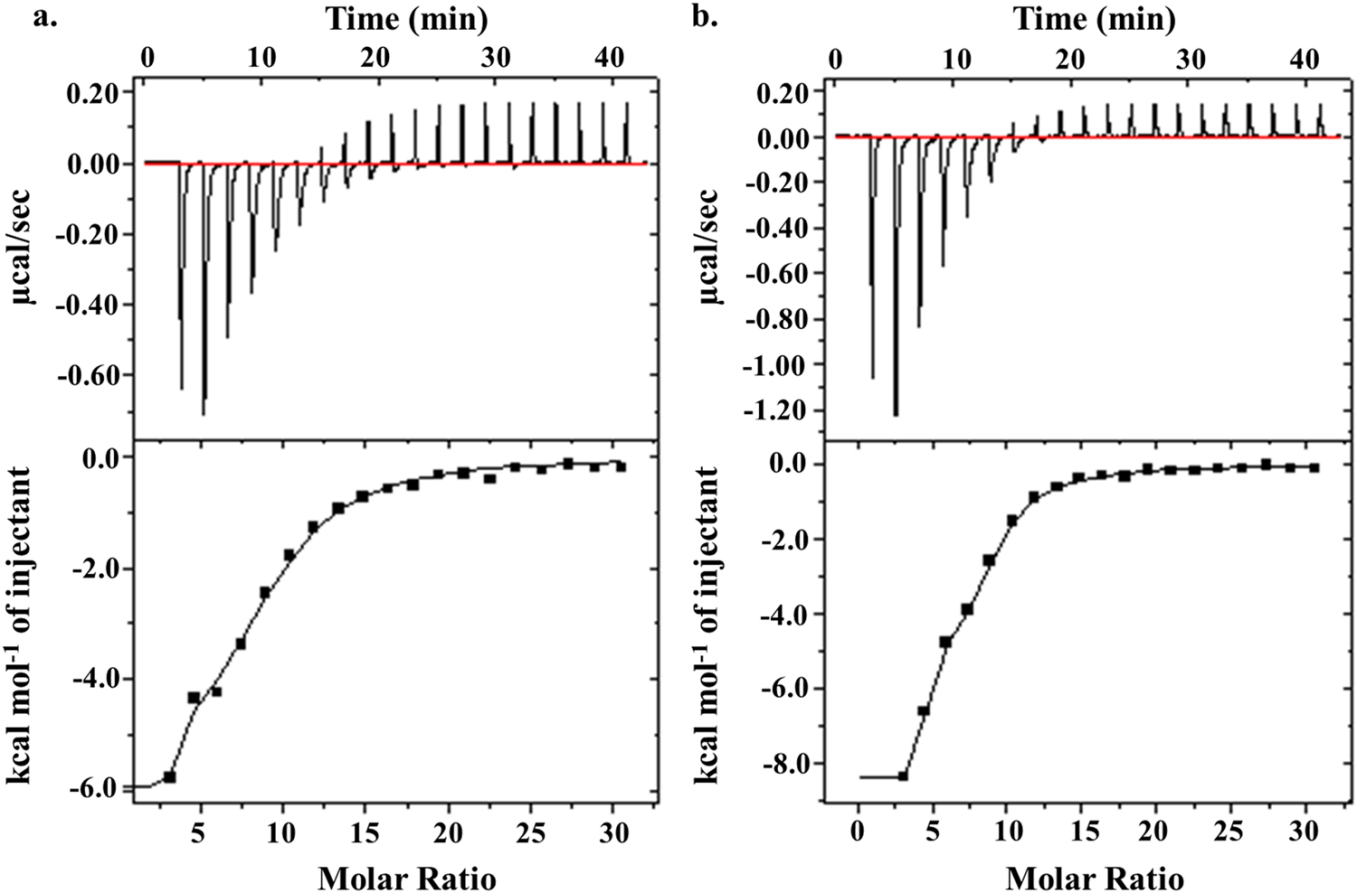
ITC thermograms of HGQs with TMPyP4. The isothermal-calorimetric thermograms of HGQ-NV-L (**a**) and HGQ-NV-G (**b**) show their binding affinities towards TMPyP4. The data for their respective mutants has been included in the supplementary section (Fig. S14.1).

Certain small molecules like TMPyP4, Braco-19, PDS, etc. possess a characteristic planar aromatic surface. The cationic moieties in them further enhance their interaction with the G-quadruplex structures. They have been extensively used to study the effect of stabilization of GQ structures in viral genes and in turn, on the growth of viruses including Ebola, Hepatitis C, HIV, etc.^27,28,46^. We, therefore, moved ahead to determine the interaction of the HGQs in NiV with TMPyP4. The interaction of TMPyP4 with the HGQs was thermodynamically favorable as observed by ITC experiments^47^. The association constants (K_a_) for HGQ-NV-L and HGQ-NV-G was noted as 1E^8^ M^−1^ and 3.32E^8^ M^−1^ (Figs. 5, S14.1) respectively which was about 100 to 1000-fold greater than the binding affinity of TMPyP4 with their respective mutants (Fig. S14.1). The ITC analysis exhibited the high binding affinity of TMPyP4 with the HGQs indicative of selective and energetically favorable interaction. Similar interaction patterns were found for the two HGQs with another known G-quadruplex binding ligand, Braco-19 (data included in Fig. S14.2).

### Stabilization of HGQs by G-quadruplex binding ligand leads to stalling of Taq DNA polymerase

**Figure 6:**
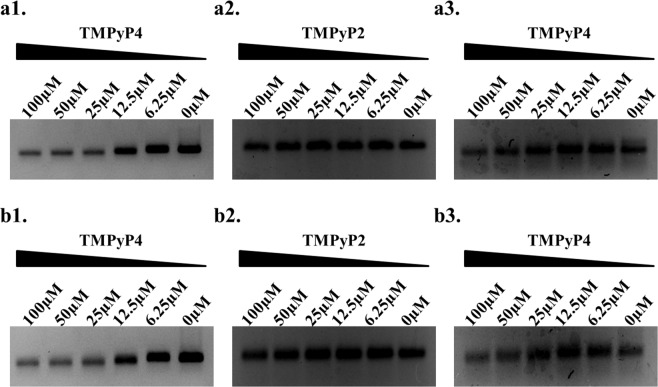
Primer extension assays. Primer extension assay shows a decrease in the intensity of band with increasing concentration of TMPyP4 for both the HGQs (a1: HGQ-NV-L with TMPyP4; a2: HGQ-NV-L with TMPyP2; a3: HGQ-NV-Lmut with TMPyP4; b1: HGQ-NV-G with TMPyP4; b2: HGQ-NV-G with TMPyP2 and b3: HGQ-NV-Gmut with TMPyP4). Full-length gels are shown in Fig. S20.

The primer extension assay is extensively used to confirm the formation of G-quadruplex in a DNA template, which acts as a road block and causes stalling of DNA polymerase. The primer extension assay was performed in the presence of increasing concentration of TMPyP4. The results show that the intensity of the product decreased in the presence of increasing concentration of TMPyP4 (Fig. 6a1,b1). These results suggest that stabilization of the HGQs by TMPyP4 causes the stalling of the Taq DNA polymerase during DNA synthesis. Additionally, in control experiments with the mutant sequences of HGQs and with TMPyP2, instead of TMPyP4, we observed no such major change in the intensity in the presence of increasing concentration of the compound (Fig. 6a3,6b3,a2,b2).

Further, the Taq DNA polymerase stop assay was performed by annealing the HGQ sequences in the 5′ end of the template with the ^32^P-labeled primer complementary end to the 3′ end of the template. In absence of any ligand, the primer extended to form the full-length product (lane 1 in Fig. S15a,b) but as the concentration of the ligand is increased, the formation of full-length product decreased due to pausing of the Taq DNA polymerase at the pause site^48^. This resulted in the generation of enhanced stop product and diminished full-length product. In case of HGQ-NV-L, at the highest concentration of Braco-19, the premature termination of the polymerase lead to nearly total inhibition of the formation of full-length product (lane 5, Fig. S15a). This data establishes the fact that ligand interaction leads to the stabilization of the HGQ sequences.

### CD melting experiments confirm the stabilization of the HGQs by G-quadruplex binding ligand

**Figure 7:**
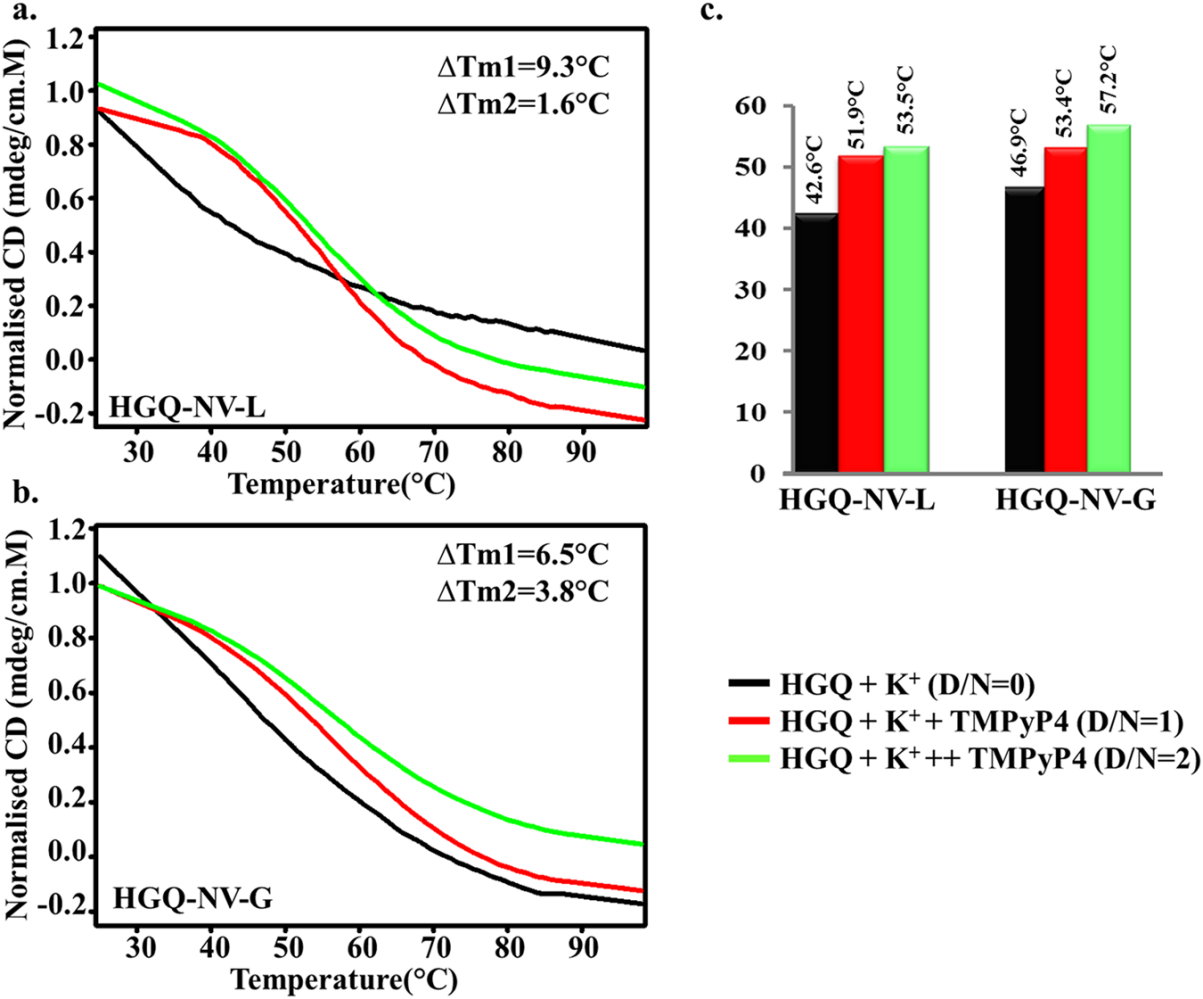
CD melting plots for the HGQs in the presence of TMPyP4 for (**a**) HGQ-NV-L and (**b**) HGQ-NV-G; (**c**) Tm values of each plot are represented in the graphical form and the values are indicated on the top of the bars.

CD melting studies also confirmed that the HGQ structures are stabilized following the addition of TMPyP4 (higher Tm value in D/N = 1) as compared to the HGQs alone. Further addition of TMPyP4, resulted in lesser ∆Tm2 (D/N2-D/N1) value as compared to ∆Tm (D/N1-D/N0) due to saturation of the binding sites on the G-quadruplex structure (Fig. 7). The stabilization of the G-quadruplex structures makes it difficult to unfold, thus leads to an increase in melting temperature. Significant change in Tm was also observed when the interactions of these HGQs with Braco-19 were evaluated through the use of CD melting experiments (data shown in Fig. S16).

### The stabilization of G-quadruplex structures results in the suppression of mTFP expression

**Figure 8:**
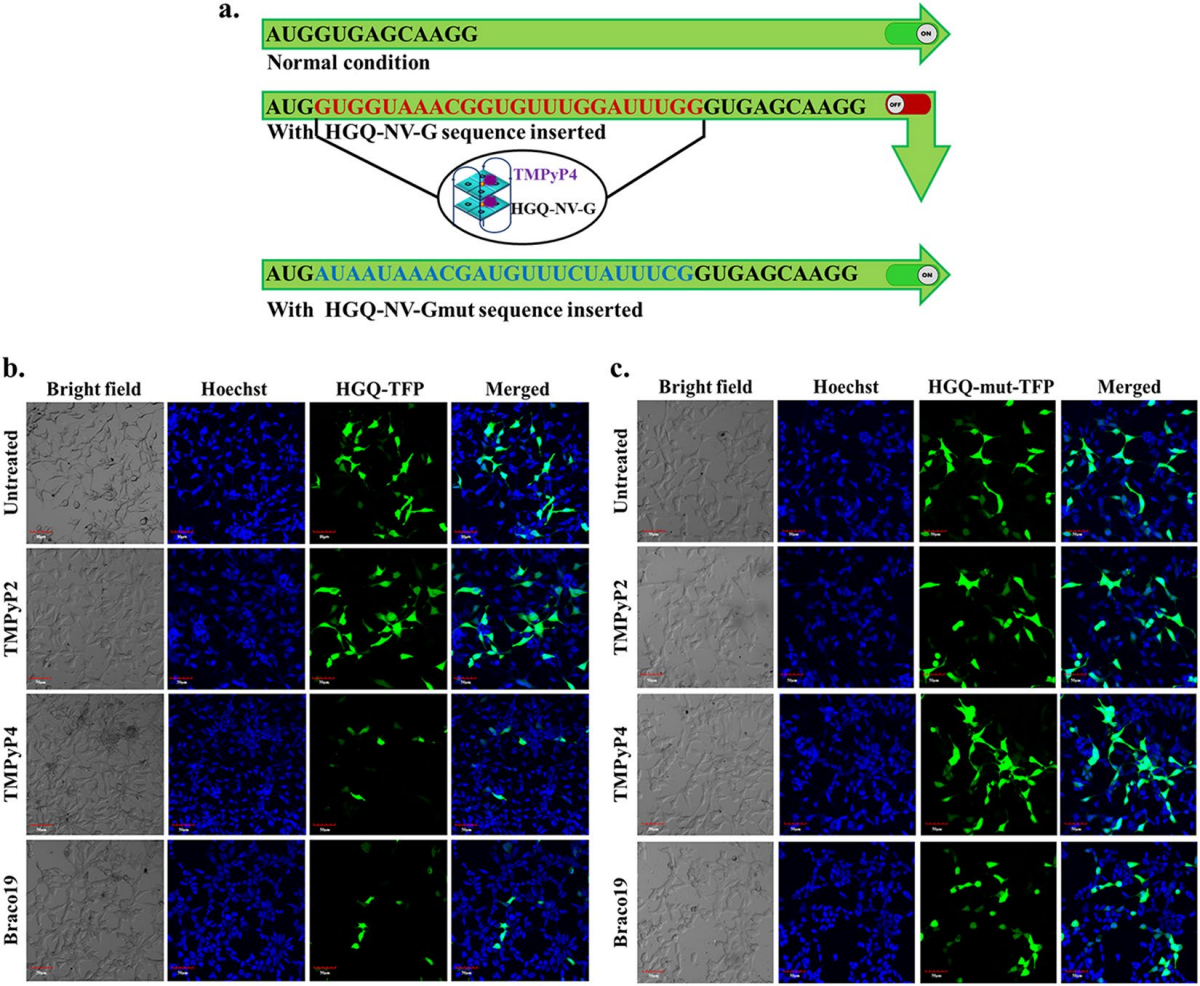
Effect of HGQ stabilization by G-quadruplex on gene expression evaluated through mTFP-based reporter assay. (**a**) Schematic representation of the cloning strategy. The HGQ of the NiV gene and its mutant sequence were cloned just after the ATG start codon of the mTFP gene. Under normal condition the mTFP gene would be translated to produce TFP protein, but in presence of stabilized G-quadruplex, the translation process will be hindered leading to attenuation of the TFP expression. (**b**) TMPyP4 and Braco-19 suppress the mTFP expression in the HEK cells as compared to the untreated or TMPyP2 treated cells due to stabilization of the HGQ structure. (**c**) No clear suppression in mTFP expression was observed for HGQ mutant sequence upon treatment with TMPyP4 or Braco-19. Scale-50 µm.

The stabilization of any secondary structure present in the protein-coding region has been reported to inhibit the translation^30,31^. The presence of a stable G-quadruplex structure within the open-reading frame of a particular gene may result in attenuation of the translation process, thus affecting the overall rate of gene expression. On this basis, we evaluated the effect of HGQs of NiV on the gene harboring them using a teal fluorescence protein (mTFP) -based reporter assay. For this, the pCAG-mTFP plasmid was engineered with HGQs which were placed upstream of the TFP codon, just after the ATG start codon (Fig. 8a). The HEK293 cells were then transfected with the reporter plasmid followed by treatment with known G-quadruplex stabilizing ligands (TMPyP4 and Braco-19). The cells from treated groups and untreated control were observed under a microscope. In the cells transfected with the HGQ-NV-G-TFP and subsequently treated with TMPyP4 or Braco-19, the TFP expression was diminished (Fig. 8b) as compared to the untreated cells and also in the cells treated with TMPyP2. The HGQ-NV-Gmut-TFP transfected HEK293 cells exhibited similar expression levels in both the treated and untreated cells (Fig. 8c). Importantly, increasing concentration of the ligand resulted in significantly higher inhibition of mTFP expression which was evident from the dose-dependent effect of TMPyP4 on the HGQ (Fig. S17).

## Discussion

With the direct visualization of G-quadruplex structures in living cells, these structures are considered to be new molecular targets for cancer therapeutics. Similarly the existence of G-quadruplex structures in the genomes of the pathogenic organisms open up new avenues for the design of antimicrobial strategies that target bacterial and viral virulence. In this study, we found the occurrence of putative G-quadruplex forming sequences in NiV genome. Using bio-informatic analysis and standard bio-physical and biochemical tools including NMR spectroscopy, EMSA, CD spectroscopy, and DMS footprinting assays, we discovered two GQ forming sequences in the essential genes of NiV genome. Studies have shown that there exist close linkage between DNA G-quadruplex forming sequences and their RNA counterparts^49^. Although the conformation of the G-quadruplex structures in DNA and RNA may vary, the RNA G-quadruplex structures are thermodynamically more stable because of the presence of the 2′-hydroxyl group of the ribose sugar^50^. Researchers have relied on G-quadruplex studies by using DNA oligonucleotides for (−)ve sense RNA containing viral genome as the DNA sequence is equally competent, robust and can provide an idea for further evaluation in their RNA analog sequences^49,51^. In line with this, we have used DNA oligonucleotides for *in vitro* assays to mimic the G-quadruplex in NiV genome. Although the Nipah virus genome contains (−)ve stranded RNA genome, during replication it uses both the genomic (−)ve and complementary (+)ve anti-genomic strand as a template. The G-quadruplex forming sequences in the genomic strand could hold potential significance in the viral replication as this can regulate the viral transcription machinery during the positive sense RNA strand synthesis. Moreover, as the (+)ve strand is predicted to have GQ forming sequences, it has direct implication in the translation of viral mRNA and template functions of anti-genomic cRNA strand. Thus, the HGQs in both the strands of the virus can act as potential targets.

G-quadruplex prediction in all isolates of NiV complete genomes revealed the existence of putative GQ sequences. The NiV isolates can be broadly classified into two clades, namely the Malaysian clade and the Bangladeshi clade^52,53^. Our prediction are also in alliance to this as some of the HGQs, for example, HGQ-NV-M1 to HGQ-NV-M8 are shown exclusively to be conserved among the Malaysian clade and are absent in the isolates of the Bangladeshi clade. While the HGQ-NV-B1 to HGQ-NV-B4 are solely present in the Bangladeshi isolates (Table S9 and S10). All the six NiV viral genes play important roles in the viral life cycle and we identifed conserved HGQs in the coding region of all the genes including matrix protein (M), nucleocapsid protein (N), phosphoprotein (P), envelope glycoprotein (G), fusion protein (F) and in the large RNA dependent RNA polymerase (L) (Table S11). While the two extensively studied HGQs, i.e. HGQ-NV-L and HGQ-NV-G are present in the L gene and G gene of the NiV genome. Both the F and G genes encode functions that are required for the viral entry. The glycoprotein aids in viral attachment preferentially to the endothelial and neuronal host cells expressing the ephrin-B2 and ephrin-B3 receptors^54,55^, while the fusion protein enables the viral coat to fuse with the plasma membrane and deliver viral genome into the host cell^56^. Subsequently, the N is essential for the viral genome replication, transcription and packaging while the viral polymerase is essential for the viral replication and transcription as this particular enzyme is absent in the host^57^. Notably, the P gene encoding phosphoprotein regulates viral pathogenesis and the M gene translates into the matrix protein. Therefore, stable G-quadruplex in any of these genes could stall the transcriptional and/or translational machinery leading to abortive RNA synthesis or non-functional protein products respectively.

Our study, for the first time revealed the existence of GQ forming sequences in the NiV genome. The sequences typically consist of two-guanine repeats which are capable of forming the two G-tetrad structures. These two G-tetrad G-quadruplexes have been shown to have a potential role in the viral life cycle^32,33^. The computational analysis of putative HGQs is ascertained by the biophysical assays. 1D ^1^H NMR exhibited the distinct imino-proton peaks in the HGQ sequence while the CD analysis showed the topology of the sequences. The EMSA experiments revealed that the HGQ-NV-L formed an intra-molecular structure and the HGQ-NV-G formed inter-molecular GQ structure in presence of ions. Further, the DMS footprinting assay confirmed the formation of stable G-quadruplex structures. In addition, the ITC analysis showed the thermodynamically favorable binding of the ligands, i.e. TMPyP4 and Braco-19 to the HGQs which was thereby supported by the thermal denaturation profile of the HGQs in absence and presence of the ligands. Moreover, Braco-19 was found to be having better stabilizing effect on the HGQs than TMPyP4 as ΔTm is more for the former. Importantly, the primer extension assay revealed that the HGQs hinder the replication process, thereby indicating their physiological significance. The reporter-based assay on the other hand provided us with a strong inception about the effect of stabilization of these HGQs in cells. Though in future, the experimental testing for this G-quadruplex binding ligands on Nipah viral infection is warranted which demands the bio-safety level 4 facilities. Taken together, our study provides new insights into structural and functional understanding of evolutionary conserved G-quadruplex forming sequences in the Nipah virus genome that will be useful for the design of the anti-viral strategies to target viral infection and virulence.

## Methods

### HGQ prediction

The available NiV complete genome sequences were retrieved from the NCBI Genome database (http://www.ncbi.nlm.nih.gov/genomes). As on 1^st^ July 2018, twelve NiV genome sequences were available in NCBI database. We searched for putative HGQs throughout these twelve complete genomes using our in-house G-quadruplex prediction tool^34^(minimum G = 2, loop length = 1 to 7), and the final search results were further confirmed by other online available G-quadruplex prediction tools like QGRS Mapper and QuadBase2^35,36^. Multiple alignments of these HGQs were performed using the Mega7.0.26^58^ tool and the consensus logo was generated using the WebLogo^59^ software available online.

All sequences were globally aligned and checked for the conservation of the HGQs among different isolates. Further, the genes were annotated in which the HGQs are present by using the NCBI Database Graphics mode.

### Sample preparation

The DNA oligonucleotide sequences were procured from Sigma Aldrich (Bangalore, India) and stock solutions of 100 μM each were prepared by dissolving the lyophilized samples in the required volume of water according to the manufacturer’s protocol. Further DNA oligomer samples for the CD and PAGE experiments were prepared by making dilutions in individual Tris-HCl buffer (pH = 7.4, 10 mM) containing 50 mM of one of the four different cations namely, K^+^, Na^+^, Li^+^, and Mg^2+^. The oligonucleotide samples were always thermally denatured by heating at 92 °C for 10 min, then allowed to reanneal slowly at room temperature overnight before each experiment.

### NMR spectroscopy

NMR Spectrometer of Model AVANCE III 400 Ascend Bruker BioSpin International AG, Switzerland equipped with a 5 mm broadband inverse (BBI) probe was used for the 1D ^1^H NMR experiments. The data obtained was further processed and analyzed by Topspin (1.3 version) software. 3 - (Trimethylsilyl) propionic-2, 2, 3, 3-D4 acid sodium salt (TSP) was taken as a reference compound. The experiments were performed in 90/10% H_2_O/D_2_O at 298 K with a 20 ppm spectral width in 1X potassium phosphate buffer (50 mM KCl concentration). The 200 µM of oligo concentration was used for the NMR experiment.

### CD spectroscopy

CD spectroscopy was performed on a Jasco J-815 Spectropolarimeter (Jasco Hachioji, Tokyo, Japan) attached with PTC-423S/15 Peltier Temperature Controller using a quartz cuvette having 1 mm optical path length and a sample volume of 200 µl. A continuous supply of nitrogen gas was maintained to prevent condensation around the cuvette due to heating. The CD spectra for the HGQs were recorded for the wavelength range of 200 nm–320 nm at 25 °C and scanning speed of 20 nm/min. The final concentration of the DNA oligomers for the CD experiment was kept 15 μM either prepared in the respective buffers (mentioned above) or water. Baseline correction was done before each experiment.

For CD melting spectroscopy, 20 μM of DNA was taken in the Tris-HCl buffer (pH = 7.4, 10 mM, K^+^ = 100 mM) and the data was collected at 290 nm for both HGQs for a temperature range of 25 °C to 98 °C. Gradually, 20 μM of TMPyP4 was added for D/N ratio 1 and 40 μM of TMPyP4 (final concentration) was added for D/N ratio 2. The data was then normalized and plotted with the help of Sigma plot 12.5. Similar method was followed for Braco-19.

### EMSA

Native polyacrylamide gel electrophoresis (PAGE) was conducted to monitor the mobility of HGQs. 25% native polyacrylamide gel was casted and run in 1X TBE (Tris-Borate- EDTA) buffer (with no cation) at 4 °C in a Bio-Rad Mini protean Tetra Vertical Electrophoresis unit maintaining the voltage at 90 V. For each set of experiment, the given HGQs was diluted to make a final concentration of 20 μM in each of the four cationic buffers (K^+^, Na^+^, Li^+^, and Mg^2+^) and loaded in the gel along with its mutant (20 μM) dissolved in K^+^ containing Tris buffer, which served as the negative control. Standard G-quadruplex forming sequence c-Myc and bcl2 was used in the same parameter and served as positive control. The gel was then stained with Ethidium Bromide and visualized with the help of ImageQuant LAS4000 (GE Healthcare, Biosciences Ltd, Sweden).

### ITC study of HGQs with ligands

The binding affinity of TMPyP4 with the HGQs and their respective mutants were studied using the MicroCal iTC200 isothermal titration calorimeter (GE Healthcare, U.S.A) at 25 **°**C. Total of 21 injections, 1.78 µl each of 1.5 mM TMPyP4 solution in 1X potassium phosphate buffer was added to the sample cell containing 9.2 µM HGQ DNA. The heat of dilution was calculated by injecting the same concentration of TMPyP4 into the sample cell containing 1X potassium phosphate buffer. It was then subtracted from the binding isotherm and then fitted using the ‘two set of sites’ model. MicroCal Origin software was used for it as well as to calculate the thermodynamic parameters. Similar protocol was followed for Braco-19 but the HGQ concentration in cell was 10 µM while Braco-19 concentration in syringe was 100 µM.

### Primer extension assays

The assay was performed using the HGQ-NV-L-F, HGQ-NV-G-F, HGQ-NV-Lmut-F and HGQ-NV-Gmut-F sequences as templates. HGQ-NV-PSA-FP and HGQ-NV-PSA-RP as set of primers for the HGQ amplification and HGQ-NV-PSA-FP with HGQ-NV-mut-PSA-RP as set of primers for the amplification of the HGQ mutants (Table S18). The final volume of the PCR reaction mixture was maintained at 25 μL which included 1X PCR Buffer, 10.0 pmol of each oligonucleotide, 2.5 units of Taq DNA polymerase and the varied concentration of TMPyP4 and TMPyP2 from 0.00 μM to 100.00 μM. The PCR cycle conditions were maintained as follows using the Masterycler Nexus Gradient: Initial denaturation at 95 °C for 2 min, followed by 30 cycles of denaturation at 95 °C for 30 s, annealing at 60 °C for 30 s, and extension at 42 °C for 2 min then a final extension at 42 ° for 20 min and finally kept at 4 °C. The amplified products were resolved on a 3% agarose gel runned in 1X TBE buffer. The gel was stained with ethidium bromide and image was captured using the ImageQuant LAS 4000.

### mTFP expression studies

The pCAG-mTFP vector was used for this experiment into which HGQ-NV-L and HGQ-NV-G sequences were cloned, just after the ATG start codon of the mTFP gene. Two set of forward primers were designed, one for the HGQ-NV-L and another for HGQ-NV-G where the 5′ end of the forward primer consisted of the EcoRI cleavage site followed by the respective HGQ sequence and the N terminus sequences of TFP in frame at the 3′ end (HGQ-NV-L-F and HGQ-NV-G-F). While the reverse primer was common for both the sequences comprising of the NheI cleavage site at the 3′end and C terminus sequence of the TFP at the 5′ end (HGQ-NV-RP) (Table S17). The forward and reverse primers for each HGQ were used to amplify the ~708 bp fragment comprising the whole mTFP gene along with the HGQ sequence, using the pCAG-mTFP plasmid as the PCR template. The fragment (desired insert) and the plasmid was individually digested with EcoRI and NheI and ligated together to generate the plasmid construct containing the HGQs, namely HGQ-NV-L-TFP and HGQ-NV-G-TFP. Similarly, the forward primers, HGQ-NV-Lmut-F and HGQ-NV-Gmut-F were used along with reverse primer HGQ-NV-RP to generate the constructs HGQ-NV-Lmut-TFP and HGQ-NV-Gmut-TFP using the same protocol. On confirmation of the constructs by sequencing, they were transfected into HEK293 cells using Lipofectamine2000 according to the manufacturer’s protocol. The transfected cells were subsequently treated with 20 µM each of TMPyP2, TMPyP4 and Braco-19 after 4 h of transfection and the expression level of the TFP were observed after 24 h of treatment using the Olympus Multi Photon Laser Scanning Microscope (FV1200MPE, IX83 Model).

## Supplementary information

Supplementary Information.
