# Supplementary material for: Identification and characterization of two conserved G-quadruplex forming motifs in the Nipah virus genome and their interaction with G-quadruplex specific ligands
Source: Sci Rep. 2020 Jan 30;10:1477. doi: 10.1038/s41598-020-58406-8 (PMC6992748; doi:10.1038/s41598-020-58406-8)

Supporting Information:

Identification and characterization of two conserved G-quadruplex forming motifs in the Nipah virus genome and their interaction with G-quadruplex specific ligands

Prativa Majee[1,∥], Subodh Kumar Mishra[1,∥], Nirali Pandya[1], Uma Shankar[1], Sanjeev Pasadi[2], K. Muniyappa[2], Debasis Nayak[1,*], and Amit Kumar[1,*]

[1]Discipline of Biosciences and Biomedical Engineering, Indian Institute of Technology Indore, Simrol, Indore 453552, India.

[2]Department of Biochemistry, Indian Institute of Science, Bangalore 560012, India

*To whom correspondence should be addresses. Tel: +91-731-2438771; Fax: +91-731-2438721; Email: amitk@iiti.ac.in

Table of Contents:

1. **Table S1:** Details of the Nipah isolates used for the prediction of HGQs (Page S3)
2. **Table S2:** The G-quadruplex prediction result for the Nipah virus using the in-house G-quadruplex prediction tool (Page S3-S10)
3. **Table S3:** The G-quadruplex prediction result using the QGRS Mapper (Page S11)
4. **Table S4:** The G-quadruplex prediction result using the QuadBase2 (Page S11)
5. **Figure S5:** The images displays the results obtained for G-quadruplex prediction in Nipah virus using the database described in the recently published paper by Lavezzo *et. al*. (Page S11-S13)
6. **Figure S6:** The multiple alignment images of the predicted HGQs in different isolates of the Nipah virus (Page S13-S18)
7. **Figure S7:** Sequence logos constructed for the analyzing the conservation of the al other HGQs predicted in NiV using the WebLogo software (Page S18-S19)
8. **Table S8:** The putative HGQs available in all the twelve isolates of Nipah virus and their respective sequences (Page S20)
9. **Table S9:** The putative HGQs available in all the eight isolates of Nipah virus belonging to Malaysian clade and their respective sequences (Page S20)
10. **Table S10:** The putative HGQs available in all the four isolates of Nipah virus belonging to Bangladeshi clade and their respective sequences (Page S20)
11. **Table S11:** The details of the predicted HGQs (Page S21)
12. **Figure S12:** CD spectra scan for the three HGQs-NV-1,2 and 3 (Page S22)
13. **Figure S13:** DMS footprinting assay (Page S23)
14. **Figure S14:** ITC binding studies of HGQs-mutants with TMPyP4 and HGQs with Braco-19 (Page S24-S25)
15. **Figure S15:** Taq DNA polymerase stop assay for both the HGQs (Page S26)
16. **Figure S16:** CD melting experiment for HGQs with Braco-19 (Page S27)
17. **Figure S17:** Dose-dependent effect of TMPyP4 on the expression of TFP plasmid bearing the HGQ sequence (Page S28)
18. **Table S18:** Primers and other oligonucleotide details (Page S29)
19. **Figure S19:** Full length gel images of Figure 3b (Page S30)
20. **Figure S20:** Full length gel images of Figure 6 (Page S31)

Supplementary Table S1: Table showing Nipah virus isolate details used for the prediction of G-quadruplex forming sequences (HGQs).

Sl. No.	Strain/ Isolate Name	Accession Number	Length of Genome (bp)	GC% Content
1	Nipah virus	NC002728.1	18246	39.88
2	Nipah virus	AF212302.2	18246	39.88
3	Nipah virus isolate UMMC1	AY029767.1	18246	39.88
4	Nipah virus isolate UMMC2	AY029768.1	18246	39.87
5	Nipah virus isolate NV/MY/99/UM-0128	AJ564623.1	18246	39.87
6	Nipah virus isolate NV/MY/99/VRI-0626	AJ627196.1	18246	39.89
7	Nipah virus isolate NV/MY/99/VRI-1413	AJ564622.1	18246	39.88
8	Nipah virus isolate NV/MY/99/VRI-2794	AJ564621.1	18246	39.87
9	Nipah virus from Bangladesh	AY988601.1	18252	40.12
10	Nipah virus isolate NIVBGD2008MANIKGONJ	JN808857.1	18252	40.31
11	Nipah virus isolate NIVBGD2008RAJBARI	JN808863.1	18252	40.31
12	Nipah virus isolate Ind-Nipah-07-FG from India	FJ513078.1	18252	40.18

Supplementary Table S2: The G-quadruplex prediction result for the Nipah virus using the in-house G-quadruplex prediction tool. {1: Nipah virus (NC002728.1); 2: Nipah Virus (AF212302.2); 3: Nipah virus isolate UMMC1 (AY029767.1); 4: Nipah virus isolate UMMC2 (AY029768.1); 5: Nipah virus isolate NV/MY/99/UM-0128 (AJ564623.1); 6: Nipah virus isolate NV/MY/99/VRI-0626 (AJ627196.1); 7: Nipah virus isolate NV/MY/99/VRI-1413 (AJ564622.1); 8: Nipah virus isolate NV/MY/99/VRI-2794 (AJ564621.1); 9: Nipah virus from Bangladesh (AY988601.1); 10: Nipah virus isolate NIVBGD2008MANIKGONJ (JN808857.1); 11: Nipah virus isolate NIVBGD2008RAJBARI (JN808863.1) and 12: Nipah virus isolate Ind-Nipah-07-FG from India (FJ513078.1)}

Along with the putative G-quadruplex sequence prediction, this tool provides us with the length of the sequences, position of the sequence in the genome i.e. the start and end position and the

cC score and the cG score. The higher cG/cC score represents higher probability to G-quadruplex structure formation. The HGQ-NV-L and HGQ-NV-G chosen for further detailed analysis are highlighted in yellow the prediction table.

1. Nipah virus (NC002728.1)

**Sense Strand**						
**#**	**Length (bp)**	**Start Position**	**End Position**	**HGQ Motifs**	**cG Score**	**cC Score**
1	20	454	473	GGAGAGGAGAGGAGACAAGG	110	10
2	15	478	492	GGAGGAGATGGAAGG	90	0
3	18	892	909	GGAAACTGGTATGGCAGG	80	20
4	24	12314	12337	GGATGAGGCTAGGATCCTGAGGGG	120	30
5	20	13151	13170	GGAGAACGGGATGGTTAAGG	100	10
6	16	15080	15095	GGCTATGGTGGGAAGG	90	10
7	20	15606	15625	GGGTTATCGGTATTGGAAGG	90	10
**Anti-sense Strand**						
**#**	**Length (bp)**	**Start Position**	**End Position**	**HGQ Motifs**	**cG Score**	**cC Score**
1	18	78	95	GGTCTTGGATTTGGAAGG	80	10
2	18	399	416	GGATAGGGTTCTAGGTGG	90	10
3	18	5573	5590	GGGGAGGTAAAGAGGAGG	110	0
4	22	8464	8485	GGTAAACGGTGTTTGGATTTGG	90	10
5	17	10334	10350	GGCTGTAGGACAGGTGG	90	20
6	25	14644	14668	GGGACTTTGGCATCGGAGTTCCTGG	100	50
7	28	14948	14975	GGCTTGAAGGTTGTCTCGGATTGATTGG	110	30

2. Nipah Virus (AF212302.2)

**Sense Strand**						
**#**	**Length (bp)**	**Start Position**	**End Position**	**HGQ Motifs**	**cG Score**	**cC Score**
1	20	454	473	GGAGAGGAGAGGAGACAAGG	110	10
2	15	478	492	GGAGGAGATGGAAGG	90	0
3	18	892	909	GGAAACTGGTATGGCAGG	80	20
4	24	12314	12337	GGATGAGGCTAGGATCCTGAGGGG	120	30
5	20	13151	13170	GGAGAACGGGATGGTTAAGG	100	10

6	16	15080	15095	GGCTATGGTGGGAAGG	90	10
7	20	15606	15625	GGGTTATCGGTATTGGAAGG	90	10
**Anti-sense Strand**						
**#**	**Length (bp)**	**Start Position**	**End Position**	**HGQ Motifs**	**cG Score**	**cC Score**
1	18	78	95	GGTCTTGGATTTGGAAGG	80	10
2	18	399	416	GGATAGGGTTCTAGGTGG	90	10
3	18	5573	5590	GGGGAGGTAAAGAGGAGG	110	0
4	22	8464	8485	GGTAAACGGTGTTTGGATTTGG	90	10
5	17	10334	10350	GGCTGTAGGACAGGTGG	90	20
6	25	14644	14668	GGGACTTTGGCATCGGAGTTCCTGG	100	50
7	28	14948	14975	GGCTTGAAGGTTGTCTCGGATTGATTGG	110	30

3. Nipah virus isolate UMMC1 (AY029767.1)

**Sense Strand**						
**#**	**Length (bp)**	**Start Position**	**End Position**	**HGQ Motifs**	**cG Score**	**cC Score**
1	20	454	473	GGAGAGGAGAGGAGACAAGG	110	10
2	15	478	492	GGAGGAGATGGAAGG	90	0
3	18	892	909	GGAAACTGGTATGGCAGG	80	20
4	24	12314	12337	GGATGAGGCTAGGATCCTGAGGGG	120	30
5	20	13151	13170	GGAGAACGGGATGGTTAAGG	100	10
6	16	15080	15095	GGCTATGGTGGGAAGG	90	10
7	20	15606	15625	GGGTTATCGGTATTGGAAGG	90	10
**Anti-sense Strand**						
**#**	**Length (bp)**	**Start Position**	**End Position**	**HGQ Motifs**	**cG Score**	**cC Score**
1	18	78	95	GGTCTTGGATTTGGAAGG	80	10
2	18	399	416	GGATAGGGTTCTAGGTGG	90	10
3	18	5573	5590	GGGGAGGTAAAGAGGAGG	110	0
4	22	8464	8485	GGTAAACGGTGTTTGGATTTGG	90	10
5	17	10334	10350	GGCTGTAGGACAGGTGG	90	20
6	25	14644	14668	GGGACTTTGGCATCGGAGTTCCTGG	100	50
7	28	14948	14975	GGCTTGAAGGTTGTCTCGGATTGATTGG	110	30

4. Nipah virus isolate UMMC2 (AY029768.1)

**Sense Strand**						
**#**	**Length (bp)**	**Start Position**	**End Position**	**HGQ Motifs**	**cG Score**	**cC Score**
1	20	454	473	GGAGAGGAGAGGAGACAAGG	110	10
2	15	478	492	GGAGGAGATGGAAGG	90	0
3	18	892	909	GGAAACTGGTATGGCAGG	80	20
4	24	12314	12337	GGATGAGGCTAGGATCCTGAGGGG	120	30
5	20	13151	13170	GGAGAACGGGATGGTTAAGG	100	10
6	16	15080	15095	GGCTATGGTGGGAAGG	90	10
7	20	15606	15625	GGGTTATCGGTATTGGAAGG	90	10
**Anti-sense Strand**						
**#**	**Length (bp)**	**Start Position**	**End Position**	**HGQ Motifs**	**cG Score**	**cC Score**
1	18	78	95	GGTCTTGGATTTGGAAGG	80	10
2	18	399	416	GGATAGGGTTCTAGGTGG	90	10
3	18	5573	5590	GGGGAGGTAAAGAGGAGG	110	0
4	22	8464	8485	GGTAAACGGTGTTTGGATTTGG	90	10
5	17	10334	10350	GGCTGTAGGACAGGTGG	90	20
6	25	14644	14668	GGGACTTTGGCATCGGAGTTCCTGG	100	50
7	28	14948	14975	GGCTTGAAGGTTGTCTCGGATTGATTGG	110	30

5. Nipah virus isolate NV/MY/99/UM-0128 (AJ564623.1)

**Sense Strand**						
**#**	**Length (bp)**	**Start Position**	**End Position**	**HGQ Motifs**	**cG Score**	**cC Score**
1	20	454	473	GGAGAGGAGAGGAGACAAGG	110	10
2	15	478	492	GGAGGAGATGGAAGG	90	0
3	18	892	909	GGAAACTGGTATGGCAGG	80	20
4	24	12314	12337	GGATGAGGCTAGGATCCTGAGGGG	120	30
5	20	13151	13170	GGAGAACGGGATGGTTAAGG	100	10
6	16	15080	15095	GGCTATGGTGGGAAGG	90	10
7	20	15606	15625	GGGTTATCGGTATTGGAAGG	90	10
**Anti-sense Strand**						
**#**	**Length (bp)**	**Start Position**	**End Position**	**HGQ Motifs**	**cG Score**	**cC Score**

1	18	78	95	GGTCTTGGATTTGGAAGG	80	10
2	18	399	416	GGATAGGGTTCTAGGTGG	90	10
3	18	5573	5590	GGGGAGGTAAAGAGGAGG	110	0
4	22	8464	8485	GGTAAACGGTGTTTGGATTTGG	90	10
5	17	10334	10350	GGCTGTAGGACAGGTGG	90	20
6	25	14644	14668	GGGACTTTGGCATCGGAGTTCCTGG	100	50
7	28	14948	14975	GGCTTGAAGGTTGTCTCGGATTGATTGG	110	30

6. Nipah virus isolate NV/MY/99/VRI-0626 (AJ627196.1)

**Sense Strand**						
**#**	**Length (bp)**	**Start Position**	**End Position**	**HGQ Motifs**	**cG Score**	**cC Score**
1	20	454	473	GGAGAGGAGAGGAGACAAGG	110	10
2	15	478	492	GGAGGAGATGGAAGG	90	0
3	18	892	909	GGAAACTGGTATGGCAGG	80	20
4	24	12314	12337	GGATGAGGCTAGGATCCTGAGGGG	120	30
5	20	13151	13170	GGAGAACGGGATGGTTAAGG	100	10
6	16	15080	15095	GGCTATGGTGGGAAGG	90	10
7	20	15606	15625	GGGTTATCGGTATTGGAAGG	90	10
**Anti-sense Strand**						
**#**	**Length (bp)**	**Start Position**	**End Position**	**HGQ Motifs**	**cG Score**	**cC Score**
1	18	74	91	GGTTGGTCTTGGATTTGG	80	10
2	18	399	416	GGATAGGGTTCTAGGTGG	90	10
3	18	5573	5590	GGGGAGGTAAAGAGGAGG	110	0
4	22	8464	8485	GGTAAACGGTGTTTGGATTTGG	90	10
5	17	10334	10350	GGCTGTAGGACAGGTGG	90	20
6	25	14644	14668	GGGACTTTGGCATCGGAGTTCCTGG	100	50
7	28	14948	14975	GGCTTGAAGGTTGTCTCGGATTGATTGG	110	30

7. Nipah virus isolate NV/MY/99/VRI-1413 (AJ564622.1)

**Sense Strand**						
**#**	**Length (bp)**	**Start Position**	**End Position**	**HGQ Motifs**	**cG Score**	**cC Score**
1	20	454	473	GGAGAGGAGAGGAGACAAGG	110	10

2	15	478	492	GGAGGAGATGGAAGG	90	0
3	18	892	909	GGAAACTGGTATGGCAGG	80	20
4	24	12314	12337	GGATGAGGCTAGGATCCTGAGGGG	120	30
5	20	13151	13170	GGAGAACGGGATGGTTAAGG	100	10
6	16	15080	15095	GGCTATGGTGGGAAGG	90	10
7	20	15606	15625	GGGTTATCGGTATTGGAAGG	90	10
**Anti-sense Strand**						
**#**	**Length (bp)**	**Start Position**	**End Position**	**HGQ Motifs**	**cG Score**	**cC Score**
1	18	78	95	GGTCTTGGATTTGGAAGG	80	10
2	18	399	416	GGATAGGGTTCTAGGTGG	90	10
3	18	5573	5590	GGGGAGGTAAAGAGGAGG	110	0
4	22	8464	8485	GGTAAACGGTGTTTGGATTTGG	90	10
5	17	10334	10350	GGCTGTAGGACAGGTGG	90	20
6	25	14644	14668	GGGACTTTGGCATCGGAGTTCCTGG	100	50
7	28	14948	14975	GGCTTGAAGGTTGTCTCGGATTGATTGG	110	30

8. Nipah virus isolate NV/MY/99/VRI-2794 (AJ564621.1)

**Sense Strand**						
**#**	**Length (bp)**	**Start Position**	**End Position**	**HGQ Motifs**	**cG Score**	**cC Score**
1	20	454	473	GGAGAGGAGAGGAGACAAGG	110	10
2	15	478	492	GGAGGAGATGGAAGG	90	0
3	18	892	909	GGAAACTGGTATGGCAGG	80	20
4	24	12314	12337	GGATGAGGCTAGGATCCTGAGGGG	120	30
5	20	13151	13170	GGAGAACGGGATGGTTAAGG	100	10
6	16	15080	15095	GGCTATGGTGGGAAGG	90	10
7	20	15606	15625	GGGTTATCGGTATTGGAAGG	90	10
**Anti-sense Strand**						
**#**	**Length (bp)**	**Start Position**	**End Position**	**HGQ Motifs**	**cG Score**	**cC Score**
1	18	78	95	GGTCTTGGATTTGGAAGG	80	10
2	18	399	416	GGATAGGGTTCTAGGTGG	90	10
3	18	5573	5590	GGGGAGGTAAAGAGGAGG	110	0
4	22	8464	8485	GGTAAACGGTGTTTGGATTTGG	90	10
5	17	10334	10350	GGCTGTAGGACAGGTGG	90	20
6	25	14644	14668	GGGACTTTGGCATCGGAGTTCCTGG	100	50

7	28	14948	14975	GGCTTGAAGGTTGTCTCGGATTGATTGG	110	30

9. Nipah virus from Bangladesh (AY988601.1)

**Sense Strand**						
**#**	**Length (bp)**	**Start Position**	**End Position**	**HGQ Motifs**	**cG Score**	**cC Score**
1	25	167	191	GGAAGAGATGGGAGGGCTAGTGCGG	140	20
2	18	892	909	GGAAACTGGCATGGCAGG	80	30
3	20	13157	13176	GGAGAACGGGATGGTTAAGG	100	10
**Anti-sense Strand**						
**#**	**Length (bp)**	**Start Position**	**End Position**	**HGQ Motifs**	**cG Score**	**cC Score**
1	18	74	91	GGTCGGTTCTGGACTTGG	80	30
2	24	5025	5048	GGAACTGAGGAAATGGATAATTGG	90	10
3	18	5573	5590	GGGGAGGTAGAGAGGAGG	120	0
4	27	8464	8490	GGTAAACGGTGTTTGGATTTGATGGGG	120	10
5	26	14640	14665	GGGATGCCTCGGGATTTTGGCATCGG	110	50

10. Nipah virus isolate NIVBGD2008MANIKGONJ (JN808857.1)

**Sense Strand**						
**#**	**Length (bp)**	**Start Position**	**End Position**	**HGQ Motifs**	**cG Score**	**cC Score**
1	25	167	191	GGAAGAGATGGGAGGGCTAGTGCGG	140	20
2	18	892	909	GGAAACTGGCATGGCAGG	80	30
3	20	13157	13176	GGAGAACGGGATGGTTAAGG	100	10
4	18	18066	18083	GGTTTCAAGGAGGTTTGG	80	10
**Anti-sense Strand**						
**#**	**Length (bp)**	**Start Position**	**End Position**	**HGQ Motifs**	**cG Score**	**cC Score**
1	18	74	91	GGTCGGTTCTGGACTTGG	80	30
2	24	5025	5048	GGAACTGAGGAAATGGATAATTGG	90	10
3	18	5573	5590	GGGGAGGTAAAGAGGAGG	110	0
4	27	8464	8490	GGTAAACGGTGTTTGGATTTGATGGGG	120	10
5	26	14640	14665	GGGATGCCTCGGGATTTTGGCATCGG	110	50

11. Nipah virus isolate NIVBGD2008RAJBARI (JN808863.1)

Sense Strand						
**#**	**Length (bp)**	**Start Position**	**End Position**	**HGQ Motifs**	**cG Score**	**cC Score**
1	25	167	191	GGAAGAGATGGGAGGGCTAGTGCGG	140	20
2	18	892	909	GGAAACTGGCATGGCAGG	80	30
3	20	13157	13176	GGAGAACGGGATGGTTAAGG	100	10
4	18	18066	18083	GGTTTCAAGGAGGTTTGG	80	10
**Anti-sense Strand**						
**#**	**Length (bp)**	**Start Position**	**End Position**	**HGQ Motifs**	**cG Score**	**cC Score**
1	18	74	91	GGTCGGTTCTGGACTTGG	80	30
2	24	5025	5048	GGAACTGAGGAAATGGATAATTGG	90	10
3	18	5573	5590	GGGGAGGTAAAGAGGAGG	110	0
4	27	8464	8490	GGTAAACGGTGTTTGGATTTGATGGGG	120	10
5	26	14640	14665	GGGATGCCTCGGGATTTTGGCATCGG	110	50

12. Nipah virus isolate Ind-Nipah-07-FG from India (FJ513078.1)

Sense Strand						
**#**	**Lengt h (bp)**	**Start Position**	**End Position**	**HGQ Motifs**	**cG Score**	**cC Score**
1	25	167	191	GGAAGAGATGGGAGGGCTAGTGCGG	140	20
2	18	892	909	GGAAACTGGCATGGCAGG	80	30
3	20	13157	13176	GGAGAACGGGATGGTTAAGG	100	10
4	18	18066	18083	GGTTTCAAGGAGGTTTGG	80	10
**Anti-sense Strand**						
**#**	**Lengt h (bp)**	**Start Position**	**End Position**	**HGQ Motifs**	**cG Score**	**cC Score**
1	18	74	91	GGTCGGTTCTGGACTTGG	80	30
2	24	5025	5048	GGAACTGAGGAAATGGATAATTGG	90	10
3	18	5573	5590	GGGGAGGTAAAGAGGAGG	110	0
4	27	8464	8490	GGTAAACGGTGTTTGGATTTGATGGGG	120	10
5	26	14640	14665	GGGATGCCTCGGGATTTTGGCATCGG	110	50

Supplementary Table S3: The G-quadruplex prediction results for the HGQ-NV-L and HGQ-NV-G of Nipah virus (NC002728.1) using the QGRS Mapper.

HGQ	Strand	Position	Length	Sequence	G-Score
HGQ-NV-L	Sense Strand	13151	20	GGAGAACGGGATGGTTAAGG	34
HGQ-NV-G	Anti-sense Strand	8464	25	GGTAAACGGTGTTTGGATTTGGTGGGG	34

Supplementary Table S4: The G-quadruplex prediction result for the HGQ-NV-L and HGQ-NV-G of Nipah virus (NC002728.1) using the QuadBase2.

HGQ	Strand	Start	End	Length	Sequence	Pattern
HGQ-NV-L	Sense Strand	13150	13171	20	GGAGAACGGGATGGTTAAGG	G2L1-7
HGQ-NV-G	Anti-sense Strand	9758	9783	25	CCACCAAATCCAAACACCGTTTACC	C2L1-7

Supplementary Figure S5: The images below display the results obtained for G-quadruplex prediction in Nipah virus genome using the database described in the recently published paper by Lavezzo *et al*. (2018) The database predicts all the G-quadruplex forming sequences in the genomes of all known human viruses. Our two conserved G-quadruplex sequence, HGQ-NV-L and HGQ-NV-G were aptly predicted by this database and their conservation was also shown to be 100%. Moreover, the predicted results were in compliance with our data as only GG islands are conserved in NiV genome.

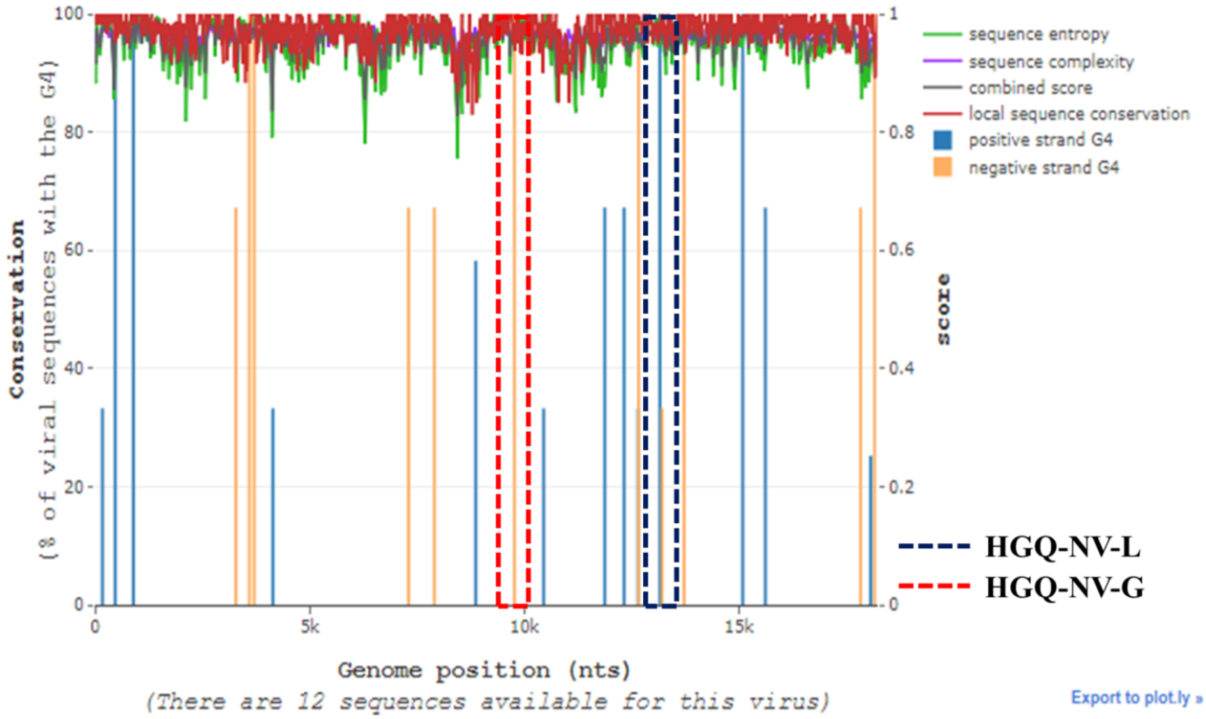

Figure S5a: The figure represents a screenshot taken for the G-quadruplex prediction results in NiV genome from the G4-virus database. The two sequences, HGQ-NV-L and HGQ-NV-G used in our study are highlighted by blue dotted box and red dotted box respectively.

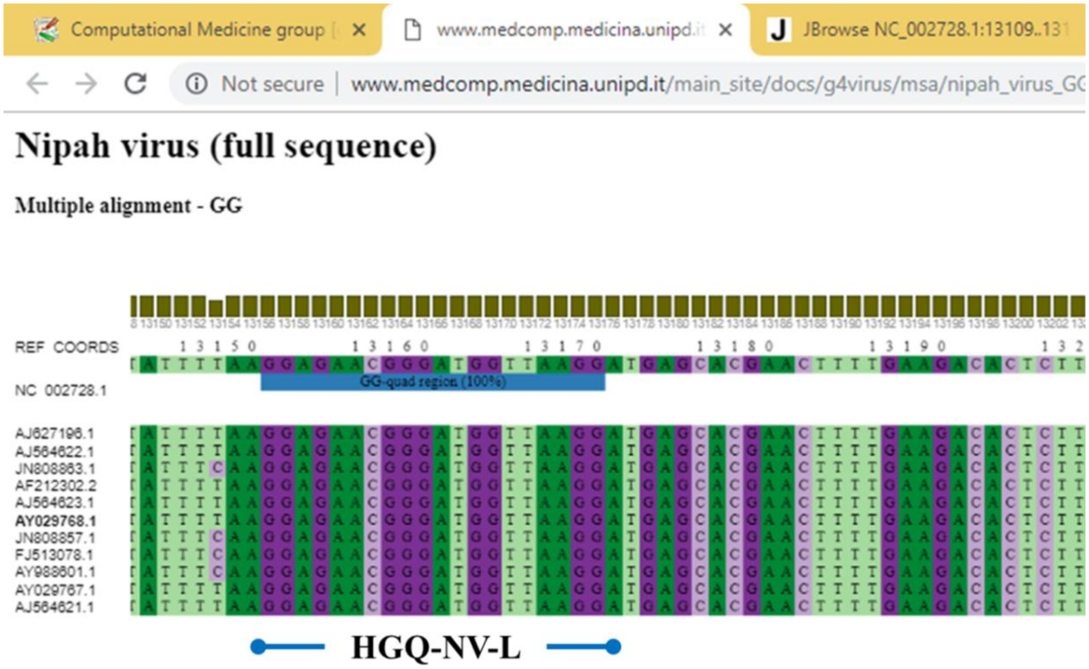

Figure S5b: The figure represents a screenshot taken for the HGQ-NV-L prediction in NiV genome using the G4-virus database. The conservation was shown to be 100%.

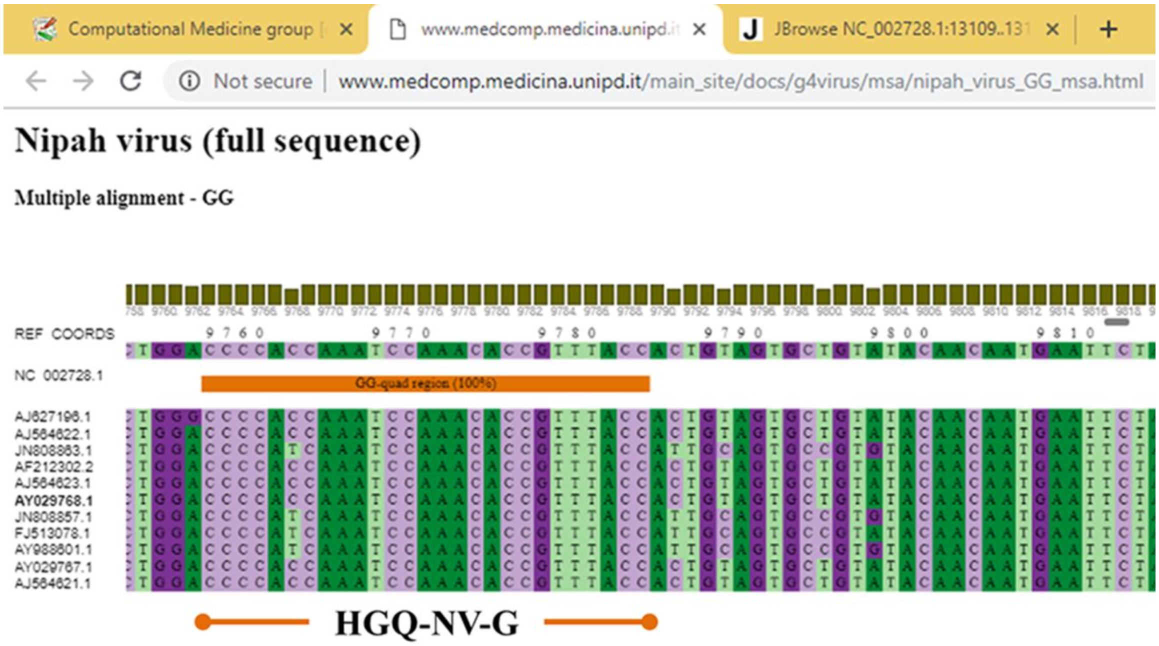

Figure S5c: The figure represents a screenshot taken for the HGQ-NV-G prediction in NiV genome using the G4-virus database. The conservation was shown to be 100% and this sequence in present in the anti-sense strand of the genome.

Supplementary Figure S6: The below images displays the multiple alignment of the predicted HGQs in different isolates of the Nipah virus prepared by using the MEGA7.0.26 software (1: HGQ-NV-L; 2: HGQ-NV-G; 3: HGQ-NV-1; 4: HGQ-NV-2; 5: HGQ-NV-3; 6: HGQ-NV-M1; 7: HGQ-NV-M2; 8: HGQ-NV-M3; 9: HGQ-NV-M4; 10: HGQ-NV-M5; 11: HGQ-NV-M6; 12: HGQ-NV-M7; 13: HGQ-NV-M8; 14: HGQ-NV-B1, 15: HGQ-NV-B2; 16: HGQ-NV-B3 and 17: HGQ-NV-B4.

1. Alignment of HGQ-NV-L present in twelve isolates of Nipah Virus

Species/Abbrv	*	*	*	*	*	*	*		*	*	*	*	*	*	*	*	*	*	*	*	*	*	*	*	*	*	*	*	*	*	*	*	*	*	*	*	*	*	*	*
1. AF212302.2_HGQ-NV-L	A	A	U	A	U	U	U	U	A	A	G	G	A	G	A	A	C	G	G	G	A	U	G	G	U	U	A	A	G	G	A	U	G	A	G	C	A	C	G	A
2. AJ564621.1_HGQ-NV-L	A	A	U	A	U	U	U	U	A	A	G	G	A	G	A	A	C	G	G	G	A	U	G	G	U	U	A	A	G	G	A	U	G	A	G	C	A	C	G	A
3. AJ564622.1_HGQ-NV-L	A	A	U	A	U	U	U	U	A	A	G	G	A	G	A	A	C	G	G	G	A	U	G	G	U	U	A	A	G	G	A	U	G	A	G	C	A	C	G	A
4. AJ564623.1_HGQ-NV-L	A	A	U	A	U	U	U	U	A	A	G	G	A	G	A	A	C	G	G	G	A	U	G	G	U	U	A	A	G	G	A	U	G	A	G	C	A	C	G	A
5. AJ627196.1_HGQ-NV-L	A	A	U	A	U	U	U	U	A	A	G	G	A	G	A	A	C	G	G	G	A	U	G	G	U	U	A	A	G	G	A	U	G	A	G	C	A	C	G	A
6. AY029767.1_HGQ-NV-L	A	A	U	A	U	U	U	U	A	A	G	G	A	G	A	A	C	G	G	G	A	U	G	G	U	U	A	A	G	G	A	U	G	A	G	C	A	C	G	A
7. AY029768.1_HGQ-NV-L	A	A	U	A	U	U	U	U	A	A	G	G	A	G	A	A	C	G	G	G	A	U	G	G	U	U	A	A	G	G	A	U	G	A	G	C	A	C	G	A
8. AY988601.1_HGQ-NV-L	A	A	U	A	U	U	U	C	A	A	G	G	A	G	A	A	C	G	G	G	A	U	G	G	U	U	A	A	G	G	A	U	G	A	G	C	A	C	G	A
9. FJ513078.1_HGQ-NV-L	A	A	U	A	U	U	U	C	A	A	G	G	A	G	A	A	C	G	G	G	A	U	G	G	U	U	A	A	G	G	A	U	G	A	G	C	A	C	G	A
10. JN808857.1_HGQ-NV-L	A	A	U	A	U	U	U	C	A	A	G	G	A	G	A	A	C	G	G	G	A	U	G	G	U	U	A	A	G	G	A	U	G	A	G	C	A	C	G	A
11. JN808863.1_HGQ-NV-L	A	A	U	A	U	U	U	C	A	A	G	G	A	G	A	A	C	G	G	G	A	U	G	G	U	U	A	A	G	G	A	U	G	A	G	C	A	C	G	A
12. NC002728.1_HGQ-NV-L	A	A	U	A	U	U	U	U	A	A	G	G	A	G	A	A	C	G	G	G	A	U	G	G	U	U	A	A	G	G	A	U	G	A	G	C	A	C	G	A

2. Alignment of HGQ-NV-G present in twelve isolates of Nipah Virus.

Species/Abbrv	*	*	*	*	*		*	*		*	*	*	*	*	*	*	*	*	*	*	*	*	*	*	*	*	*	*	*	*	*		*	*	*	*	*		*	*	*	*
1. AF212302.2_HGQ-NV-G	G	C	A	C	U	A	C	A	G	U	G	G	U	A	A	A	C	G	G	U	G	U	U	U	G	G	A	U	U	U	G	G	U	G	G	G	G	U	C	C	A	G
2. AJ564621.1_HGQ-NV-G	G	C	A	C	U	A	C	A	G	U	G	G	U	A	A	A	C	G	G	U	G	U	U	U	G	G	A	U	U	U	G	G	U	G	G	G	G	U	C	C	A	G
3. AJ564622.1_HGQ-NV-G	G	C	A	C	U	A	C	A	G	U	G	G	U	A	A	A	C	G	G	U	G	U	U	U	G	G	A	U	U	U	G	G	U	G	G	G	G	U	C	C	A	G
4. AJ564623.1_HGQ-NV-G	G	C	A	C	U	A	C	A	G	U	G	G	U	A	A	A	C	G	G	U	G	U	U	U	G	G	A	U	U	U	G	G	U	G	G	G	G	U	C	C	A	G
5. AJ627196.1_HGQ-NV-G	G	C	A	C	U	A	C	A	G	U	G	G	U	A	A	A	C	G	G	U	G	U	U	U	G	G	A	U	U	U	G	G	U	G	G	G	G	C	C	C	A	G
6. AY029767.1_HGQ-NV-G	G	C	A	C	U	A	C	A	G	U	G	G	U	A	A	A	C	G	G	U	G	U	U	U	G	G	A	U	U	U	G	G	U	G	G	G	G	U	C	C	A	G
7. AY029768.1_HGQ-NV-G	G	C	A	C	U	A	C	A	G	U	G	G	U	A	A	A	C	G	G	U	G	U	U	U	G	G	A	U	U	U	G	G	U	G	G	G	G	U	C	C	A	G
8. AY988601.1_HGQ-NV-G	G	C	A	C	U	G	C	A	A	U	G	G	U	A	A	A	C	G	G	U	G	U	U	U	G	G	A	U	U	U	G	A	U	G	G	G	G	U	C	C	A	G
9. FJ513078.1_HGQ-NV-G	G	C	A	C	U	G	C	A	A	U	G	G	U	A	A	A	C	G	G	U	G	U	U	U	G	G	A	U	U	U	G	A	U	G	G	G	G	U	C	C	A	G
10. JN808857.1_HGQ-NV-G	G	C	A	C	U	G	C	A	A	U	G	G	U	A	A	A	C	G	G	U	G	U	U	U	G	G	A	U	U	U	G	A	U	G	G	G	G	U	C	C	A	G
11. JN808863.1_HGQ-NV-G	G	C	A	C	U	G	C	A	A	U	G	G	U	A	A	A	C	G	G	U	G	U	U	U	G	G	A	U	U	U	G	A	U	G	G	G	G	U	C	C	A	G
12. NC002728.1_HGQ-NV-G	G	C	A	C	U	A	C	A	G	U	G	G	U	A	A	A	C	G	G	U	G	U	U	U	G	G	A	U	U	U	G	G	U	G	G	G	G	U	C	C	A	G

3. Alignment of HGQ-NV-1 present in twelve isolates of Nipah Virus.

Species/Abbrv	*	*	*	*	*	*	*	*	*	*	*	*	*	*	*	*	*	*	*		*	*	*	*	*	*	*	*	*	*	*	*	*	*	*	*	*	*
1. AF212302.2_HGQ-NV-1	A	C	U	A	U	G	U	C	G	A	G	G	A	A	A	C	U	G	G	U	A	U	G	G	C	A	G	G	A	U	U	C	U	U	C	G	C	A
2. AJ564621.1_HGQ-NV-1	A	C	U	A	U	G	U	C	G	A	G	G	A	A	A	C	U	G	G	U	A	U	G	G	C	A	G	G	A	U	U	C	U	U	C	G	C	A
3. AJ564622.1_HGQ-NV-1	A	C	U	A	U	G	U	C	G	A	G	G	A	A	A	C	U	G	G	U	A	U	G	G	C	A	G	G	A	U	U	C	U	U	C	G	C	A
4. AJ564623.1_HGQ-NV-1	A	C	U	A	U	G	U	C	G	A	G	G	A	A	A	C	U	G	G	U	A	U	G	G	C	A	G	G	A	U	U	C	U	U	C	G	C	A
5. AJ627196.1_HGQ-NV-1	A	C	U	A	U	G	U	C	G	A	G	G	A	A	A	C	U	G	G	U	A	U	G	G	C	A	G	G	A	U	U	C	U	U	C	G	C	A
6. AY029767.1_HGQ-NV-1	A	C	U	A	U	G	U	C	G	A	G	G	A	A	A	C	U	G	G	U	A	U	G	G	C	A	G	G	A	U	U	C	U	U	C	G	C	A
7. AY029768.1_HGQ-NV-1	A	C	U	A	U	G	U	C	G	A	G	G	A	A	A	C	U	G	G	U	A	U	G	G	C	A	G	G	A	U	U	C	U	U	C	G	C	A
8. AY988601.1_HGQ-NV-1	A	C	U	A	U	G	U	C	G	A	G	G	A	A	A	C	U	G	G	C	A	U	G	G	C	A	G	G	A	U	U	C	U	U	C	G	C	A
9. FJ513078.1_HGQ-NV-1	A	C	U	A	U	G	U	C	G	A	G	G	A	A	A	C	U	G	G	C	A	U	G	G	C	A	G	G	A	U	U	C	U	U	C	G	C	A
10. JN808857.1_HGQ-NV-1	A	C	U	A	U	G	U	C	G	A	G	G	A	A	A	C	U	G	G	C	A	U	G	G	C	A	G	G	A	U	U	C	U	U	C	G	C	A
11. JN808863.1_HGQ-NV-1	A	C	U	A	U	G	U	C	G	A	G	G	A	A	A	C	U	G	G	C	A	U	G	G	C	A	G	G	A	U	U	C	U	U	C	G	C	A
12. NC002728.1_HGQ-NV-1	A	C	U	A	U	G	U	C	G	A	G	G	A	A	A	C	U	G	G	U	A	U	G	G	C	A	G	G	A	U	U	C	U	U	C	G	C	A

4. Alignment of HGQ-NV-2 present in twelve isolates of Nipah Virus.

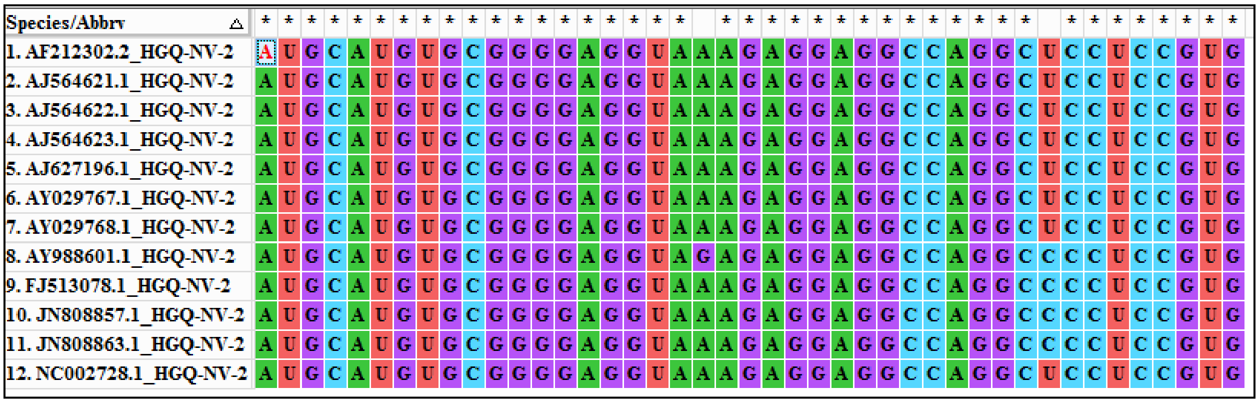

5. Alignment of HGQ-NV-3 present in twelve isolates of Nipah Virus.

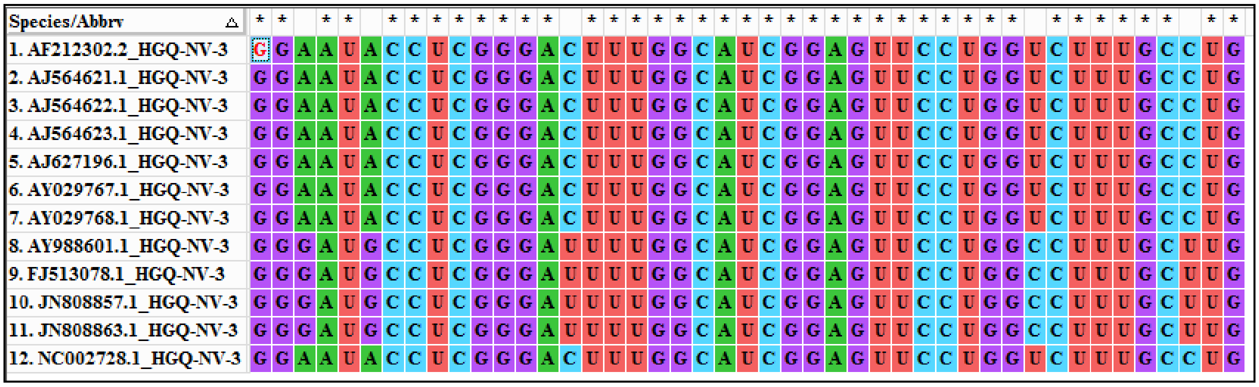

6. Alignment of HGQ-NV-M1 present in eight isolates of Nipah Virus (Malaysian clade).

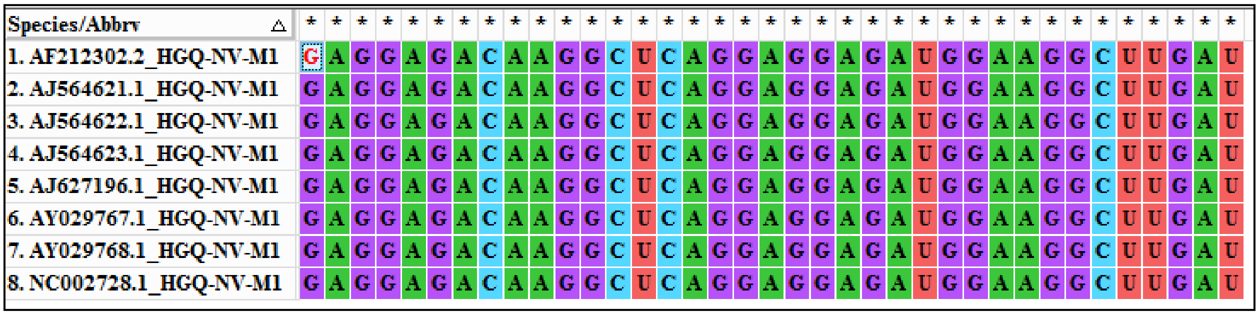

7. Alignment of HGQ-NV-M2 present in eight isolates of Nipah Virus (Malaysian clade).

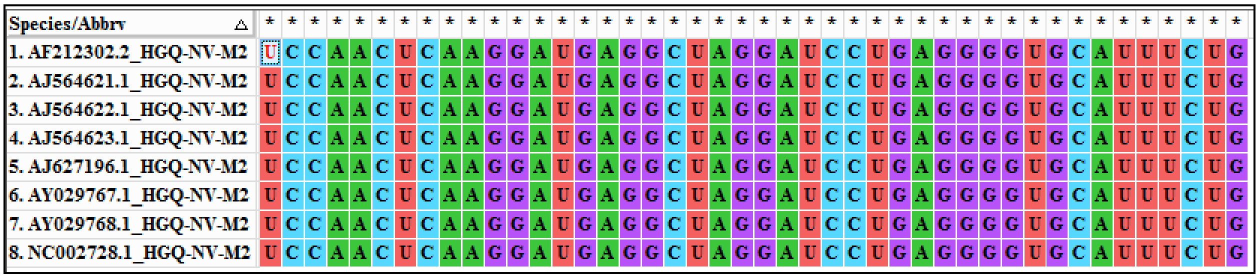

8. Alignment of GQS-NV-M3 present in eight isolates of Nipah Virus (Malaysian clade).

Species/Abbrv	* *
1. AF212302.2_HGQ-NV-M3	A U G C A C U U G A G G C U A U G G U G G G A A G G U A U A U A A C A G
2. AJ564621.1_HGQ-NV-M3	A U G C A C U U G A G G C U A U G G U G G G A A G G U A U A U A A C A G
3. AJ564622.1_HGQ-NV-M3	A U G C A C U U G A G G C U A U G G U G G G A A G G U A U A U A A C A G
4. AJ564623.1_HGQ-NV-M3	A U G C A C U U G A G G C U A U G G U G G G A A G G U A U A U A A C A G
5. AJ627196.1_HGQ-NV-M3	A U G C A C U U G A G G C U A U G G U G G G A A G G U A U A U A A C A G
6. AY029767.1_HGQ-NV-M3	A U G C A C U U G A G G C U A U G G U G G G A A G G U A U A U A A C A G
7. AY029768.1_HGQ-NV-M3	A U G C A C U U G A G G C U A U G G U G G G A A G G U A U A U A A C A G
8. NC002728.1_HGQ-NV-M3	A U G C A C U U G A G G C U A U G G U G G G A A G G U A U A U A A C A G

9. Alignment of HGQ-NV-M4 present in eight isolates of Nipah Virus (Malaysian clade).

Species/Abbrv	* *
1. AF212302.2_HGQ-NV-M4	A A U G C U A U U A G G G U U A U C G G U A U U G G A A G G U A A A U U C A G A
2. AJ564621.1_HGQ-NV-M4	A A U G C U A U U A G G G U U A U C G G U A U U G G A A G G U A A A U U C A G A
3. AJ564622.1_HGQ-NV-M4	A A U G C U A U U A G G G U U A U C G G U A U U G G A A G G U A A A U U C A G A
4. AJ564623.1_HGQ-NV-M4	A A U G C U A U U A G G G U U A U C G G U A U U G G A A G G U A A A U U C A G A
5. AJ627196.1_HGQ-NV-M4	A A U G C U A U U A G G G U U A U C G G U A U U G G A A G G U A A A U U C A G A
6. AY029767.1_HGQ-NV-M4	A A U G C U A U U A G G G U U A U C G G U A U U G G A A G G U A A A U U C A G A
7. AY029768.1_HGQ-NV-M4	A A U G C U A U U A G G G U U A U C G G U A U U G G A A G G U A A A U U C A G A
8. NC002728.1_HGQ-NV-M4	A A U G C U A U U A G G G U U A U C G G U A U U G G A A G G U A A A U U C A G A

10. Alignment of HGQ-NV-M5 present in eight isolates of Nipah Virus (Malaysian clade).

Species/Abbrv	* *
1. AF212302.2_HGQ-NV-M5	U G U U G U C C U U G G C U U G A A G G U U G U C U C G G A U U G A U U G G C C U U C G A U U G
2. AJ564621.1_HGQ-NV-M5	U G U U G U C C U U G G C U U G A A G G U U G U C U C G G A U U G A U U G G C C U U C G A U U G
3. AJ564622.1_HGQ-NV-M5	U G U U G U C C U U G G C U U G A A G G U U G U C U C G G A U U G A U U G G C C U U C G A U U G
4. AJ564623.1_HGQ-NV-M5	U G U U G U C C U U G G C U U G A A G G U U G U C U C G G A U U G A U U G G C C U U C G A U U G
5. AJ627196.1_HGQ-NV-M5	U G U U G U C C U U G G C U U G A A G G U U G U C U C G G A U U G A U U G G C C U U C G A U U G
6. AY029767.1_HGQ-NV-M5	U G U U G U C C U U G G C U U G A A G G U U G U C U C G G A U U G A U U G G C C U U C G A U U G
7. AY029768.1_HGQ-NV-M5	U G U U G U C C U U G G C U U G A A G G U U G U C U C G G A U U G A U U G G C C U U C G A U U G
8. NC002728.1_HGQ-NV-M5	U G U U G U C C U U G G C U U G A A G G U U G U C U C G G A U U G A U U G G C C U U C G A U U G

11. Alignment of HGQ-NV-M6 present in eight isolates of Nipah Virus (Malaysian clade).

Species/Abbrv	* *
1. AF212302.2_HGQ-NV-M6	U A C C G A G U A C G G C U G U A G G A C A G G U G G U G U U G U C A A U
2. AJ564621.1_HGQ-NV-M6	U A C C G A G U A C G G C U G U A G G A C A G G U G G U G U U G U C A A U
3. AJ564622.1_HGQ-NV-M6	U A C C G A G U A C G G C U G U A G G A C A G G U G G U G U U G U C A A U
4. AJ564623.1_HGQ-NV-M6	U A C C G A G U A C G G C U G U A G G A C A G G U G G U G U U G U C A A U
5. AJ627196.1_HGQ-NV-M6	U A C C G A G U A C G G C U G U A G G A C A G G U G G U G U U G U C A A U
6. AY029767.1_HGQ-NV-M6	U A C C G A G U A C G G C U G U A G G A C A G G U G G U G U U G U C A A U
7. AY029768.1_HGQ-NV-M6	U A C C G A G U A C G G C U G U A G G A C A G G U G G U G U U G U C A A U
8. NC002728.1_HGQ-NV-M6	U A C C G A G U A C G G C U G U A G G A C A G G U G G U G U U G U C A A U

12. Alignment of GQS-NV-M7 present in eight isolates of Nipah Virus (Malaysian clade).

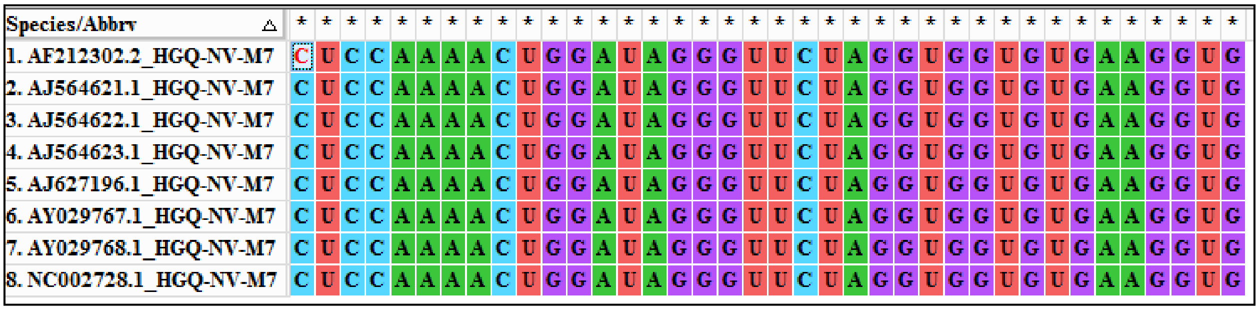

13. Alignment of HGQ-NV-M8 present in eight strains of Nipah Virus (Malaysian clade).

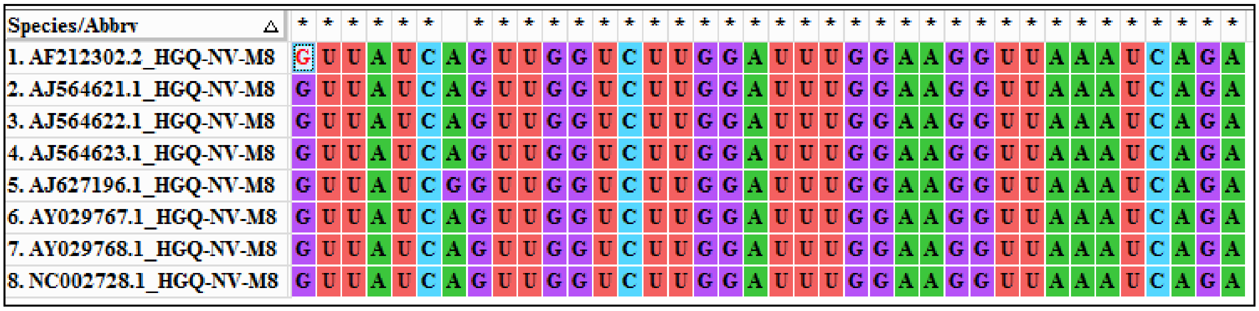

14. Alignment of HGQ-NV-B1 present in four isolates of Nipah Virus (Bangladeshi clade).

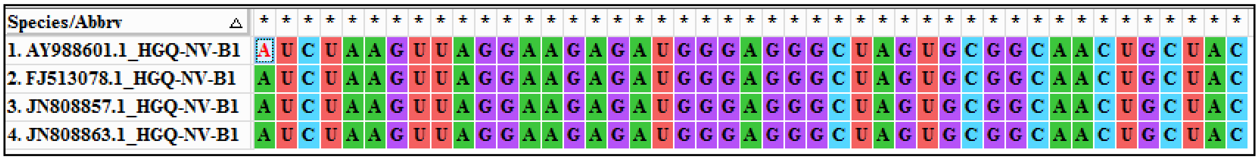

15. Alignment of HGQ-NV-B2 present in four isolates of Nipah Virus (Bangladeshi clade).

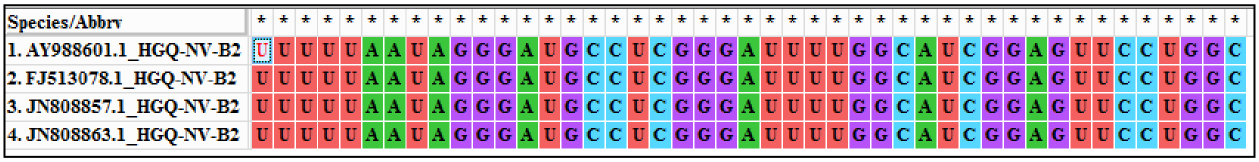

16. Alignment of HGQ-NV-B3 present in four isolates of Nipah Virus (Bangladeshi clade).

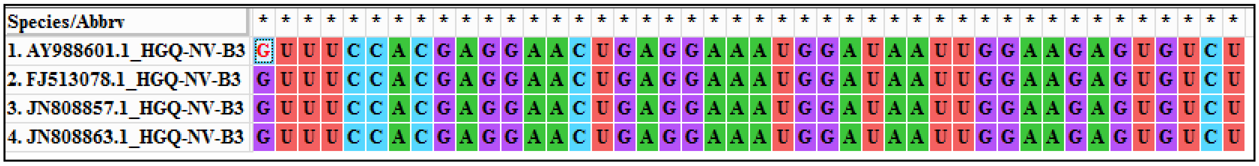

17. Alignment of HGQ-NV-B4 present in four isolates of Nipah Virus (Bangladeshi clade).

Species/Abbrv	*		*	*	*	*	*	*	*	*	*	*	*	*	*	*	*	*	*	*	*	*	*	*	*	*	*	*	*	*	*	*	*	*	*	*	*	*
1. AY988601.1_HGQ-NV-B4	A	U	A	A	A	U	U	G	U	C	G	G	U	C	G	G	U	U	C	U	G	G	A	C	U	U	G	G	A	A	G	A	U	C	A	A	A	U
2. FJ513078.1_HGQ-NV-B4	A	C	A	A	A	U	U	G	U	C	G	G	U	C	G	G	U	U	C	U	G	G	A	C	U	U	G	G	A	A	G	A	U	C	A	A	A	U
3. JN808857.1_HGQ-NV-B4	A	C	A	A	A	U	U	G	U	C	G	G	U	C	G	G	U	U	C	U	G	G	A	C	U	U	G	G	A	A	G	A	U	C	A	A	A	U
4. JN808863.1_HGQ-NV-B4	A	C	A	A	A	U	U	G	U	C	G	G	U	C	G	G	U	U	C	U	G	G	A	C	U	U	G	G	A	A	G	A	U	C	A	A	A	U

Supplementary Figure S7: Sequence logos constructed for the analyzing the conservation of the HGQs predicted in NiV using the WebLogo software. While highly conserved nucleotides are represented by a bigger letter, the large variable nucleotides represented by smaller or non-existent letters. The Sequences are either conserved in the Malaysian clade or the Bangladeshi clade and therefore, are not globally conserved. At least, one of the guanine residues essential for the G-quadruplex sequence is not globally conserved.

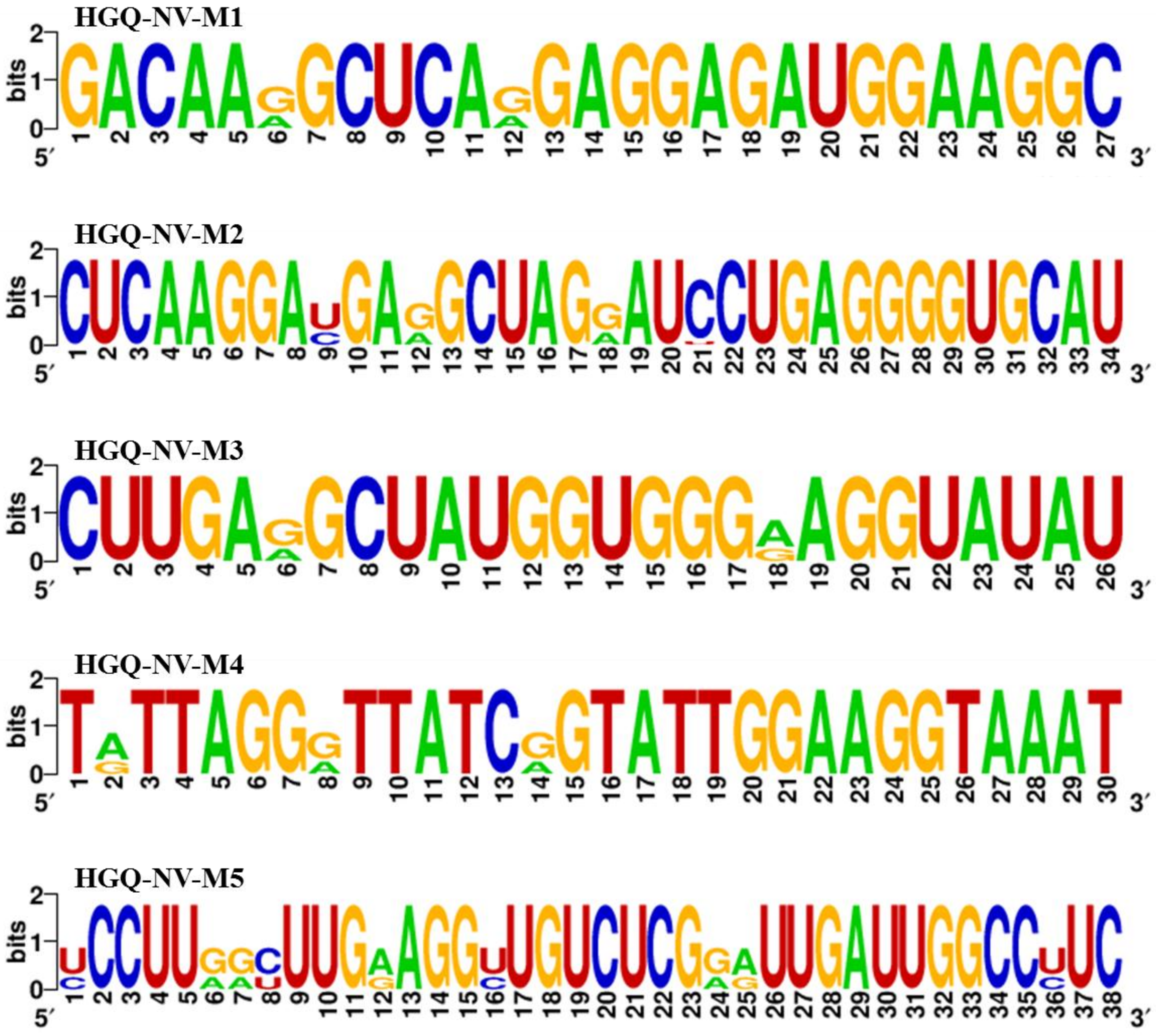

HGQ-NV-M6

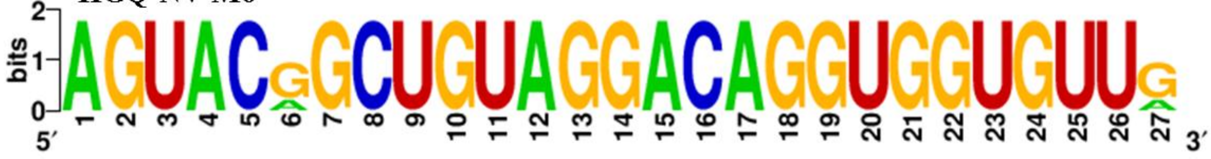

HGQ-NV-M7

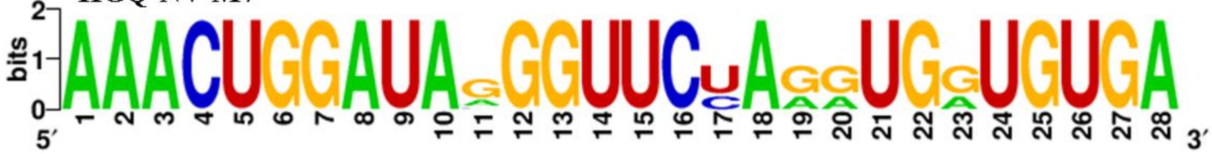

HGQ-NV-M8

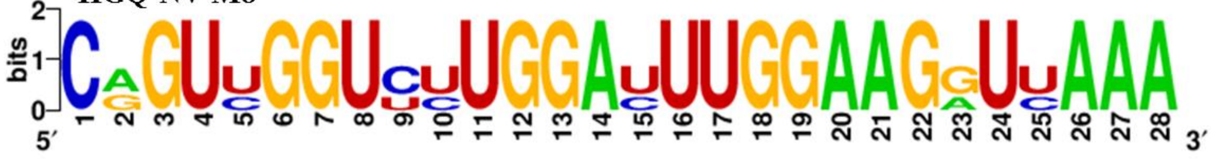

HGQ-NV-B1

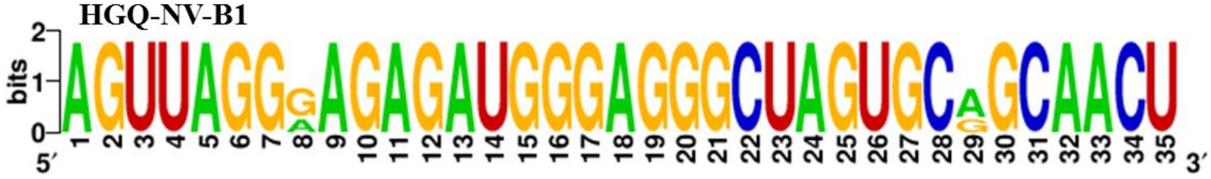

HGQ-NV-B2

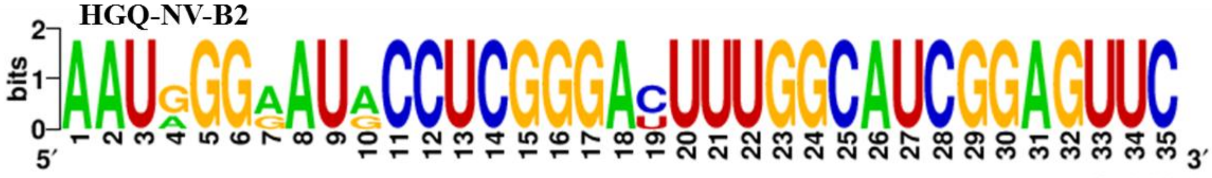

HGQ-NV-B3

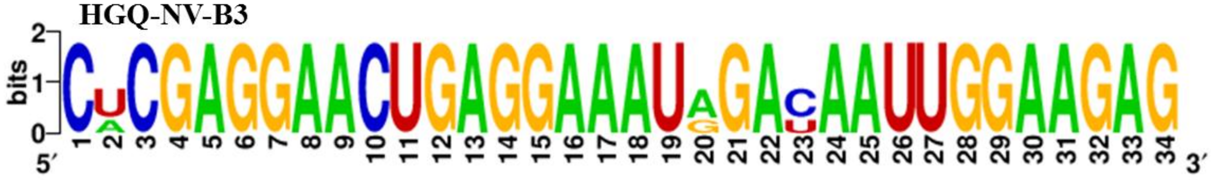

HGQ-NV-B4

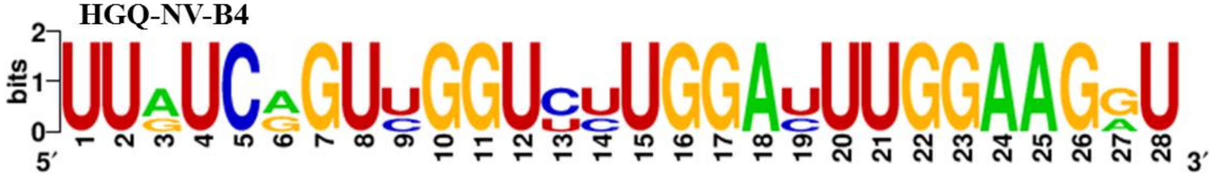

Supplementary Table S8: Table showing the putative G-quadruplex forming sequences (HGQs) available in all the twelve isolates of Nipah virus and their respective sequences.

Sl No.	HGQ Name	HGQ Sequence
1	HGQ-NV-L	UUUAAGGAGAACGGGAUGGUUAAGGAUGAG
2	HGQ-NV-G	ACAGUGGUAAACGGUGUUUGGAUUUGGUGGGGUCCAG
3	HGQ-NV-1	GUCGAGGAAACUGGUAUGGCAGGAUUCU
4	HGQ-NV-2	UGUGCGGGGAGGUAAAGAGGAGGCCAGGCUCCU
5	HGQ-NV-3	ACCUCGGGACUUUGGCAUCGGAGUUCCUGGUCUUU

Supplementary Table S9: Table showing the putative G-quadruplex forming sequences (HGQs) available in all the eight isolates of Nipah virus belonging to Malaysian clade and their respective sequences.

Sl No.	HGQ Name	HGQ Sequence
1	HGQ-NV-M1	GACAAGGCUCAGGAGGAGAUGGAAGGC
2	HGQ-NV-M2	CUCAAGGAUGAGGCUAGGAUCCUGAGGGGUGCAU
3	HGQ-NV-M3	CUUGAGGCUAUGGUGGGAAGGUAUAU
4	HGQ-NV-M4	UAUUAGGGUUAUCGGUAUUGGAAGGUAAAU
5	HGQ-NV-M5	UCCUUGGCUUGAAGGUUGUCUCGGAUUGAUUGGCCUUC
6	HGQ-NV-M6	AGUACGGCUGUAGGACAGGUGGUGUUG
7	HGQ-NV-M7	AAACUGGAUAGGGUUCUAGGUGGUGUGA
8	HGQ-NV-M8	CAGUUGGUCUUGGAUUUGGAAGGUUAAA

Supplementary Table S10: Table showing the putative G-quadruplex forming sequences (HGQs) available in all the four isolates of Nipah virus belonging to Bangladesh clade and their respective sequences.

Sl No.	HGQ Name	HGQ Sequence
1	HGQ-NV-B1	AGUUAGGAAGAGAUGGGAGGGCUAGUGCGGCAACU
2	HGQ-NV-B2	UAAUAGGGAUGCCUCGGGAUUUUGGCAUCGGAGUUC
3	HGQ-NV-B3	CACGAGGAACUGAGGAAAUGGAUAAUUGGAAGAG
4	HGQ-NV-B4	UUGUCGGUCGGUUCUGGACUUGGAAGAU

Supplementary Table S11: Table showing the details of the predicted HGQs; the strand of Nipah virus in which it is present, the genomic location of the HGQs in the virus and the P value which represents the percentage of isolates in which the particular HGQs in conserved among the total twelve viral isolates.

Sl No.	GQS Name	Strand Orientation	Gene Involved	P Value
1	HGQ-NV-L	Sense Strand	RNA dependent RNA Polymerase	100%
2	HGQ-NV-G	Anti-sense Strand	Attachment Glycoprotein	100%
3	HGQ-NV-1	Sense Strand	Nucleocapsid Protein	100%
4	HGQ-NV-2	Anti-sense Strand	Matrix Protein	100%
5	HGQ-NV-3	Anti-sense Strand	RNA dependent RNA Polymerase	100%
6	HGQ-NV-M1	Sense Strand	Nucleocapsid Protein	66.7%
7	HGQ-NV-M2	Sense Strand	RNA dependent RNA Polymerase	66.7%
8	HGQ-NV-M3	Sense Strand	RNA dependent RNA Polymerase	66.7%
9	HGQ-NV-M4	Sense Strand	RNA dependent RNA Polymerase	66.7%
10	HGQ-NV-M5	Anti-sense Strand	Phosphoprotein	66.7%
11	HGQ-NV-M6	Anti-sense Strand	Fusion Protein	66.7%
12	HGQ-NV-M7	Anti-sense Strand	RNA dependent RNA Polymerase	66.7%
13	HGQ-NV-M8	Anti-sense Strand	RNA dependent RNA Polymerase	66.7%
14	HGQ-NV-B1	Sense Strand	Nucleocapsid Protein	33.3%
15	HGQ-NV-B2	Anti-sense Strand	Phosphoprotein	33.3%
16	HGQ-NV-B3	Anti-sense Strand	RNA dependent RNA Polymerase	33.3%
17	HGQ-NV-B4	Anti-sense Strand	RNA dependent RNA Polymerase	33.3%

Supplementary Figure S12: CD spectra scans for the three HGQs: a. HGQ-NV-1, b. HGQ-NV-2 and c. HGQ-NV-3, in Tris-HCl buffer containing either K^+, Na^+, Mg^{2+} or Li^+ along with the mutant of the respective GQS. The HGQ-NV-1, HGQ-NV-2 and HGQ-NV-3 are conserved in all the Nipah virus isolates but they did not show the typical signature pattern of G-quadruplex structures rather it showed a positive peak at ~280 nm. So these three HGQs were excluded from our further studies.

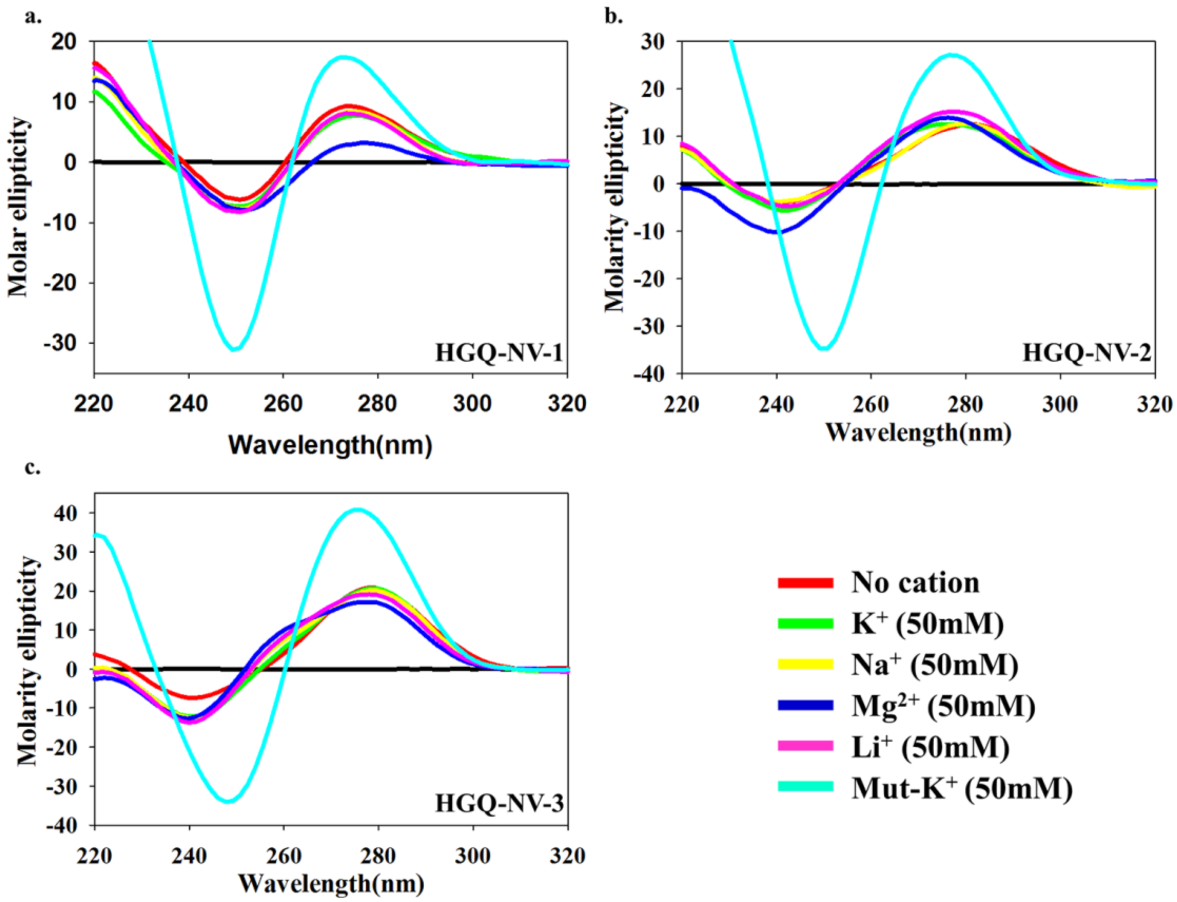

Supplementary Figure S13: DMS footprinting assay

The DNA substrates i.e. HGQ-NV-L-DMS and HGQ-NV-G-DMS (refer Table S18) were labeled at 5' end with the [γ-^{32}P]ATP (Perkin-Elmer) using polynucleotide kinase (NEB). They were then subjected to Tris-HCl buffer (100 mM KCl , pH 7.4) for GQ formation and heating at 95°C for 5 min followed by slow cooling. The single-stranded DNA substrates were dissolved in Tris-HCl buffer without KCl, heated at 95°C for 5 min and snap-chilled on ice for 10 min. All the DNA substrates were then gel-purified. For the DMS footprinting assay, the purified DNA substrates, both single-stranded and GQ, were treated with 1% DMS for 5 min at room temperature and then the reaction were terminated by adding excess of calf-thymus DNA. The DNA was retrieved by ethanol precipitation which was further treated with 10% piperidine and heated at 95°C for 30 min. The DNA fragments were again ethanol precipitated thrice to remove piperidine residues and then resolved on 17% denaturing urea polyacrylamide gels at 1600V for 2.5 h in TBE buffer. The dried gels were then exposed a phosphorimaging screen and images were captured using the Fuji FLA-5000 phosphorImager.

(a) HGQ-NV-L: 5' AAGGAGAACGGGATGGTTAAGGAT 3'

3 4 5 9 10 11 14 15 20 21

(b) HGQ-NV-G: 5' GTGGTAAACGGTGTTTGGATTTGGTGGGGTC 3'

1 3 4 10 11 13 17 18 23 24 26 27 28 29

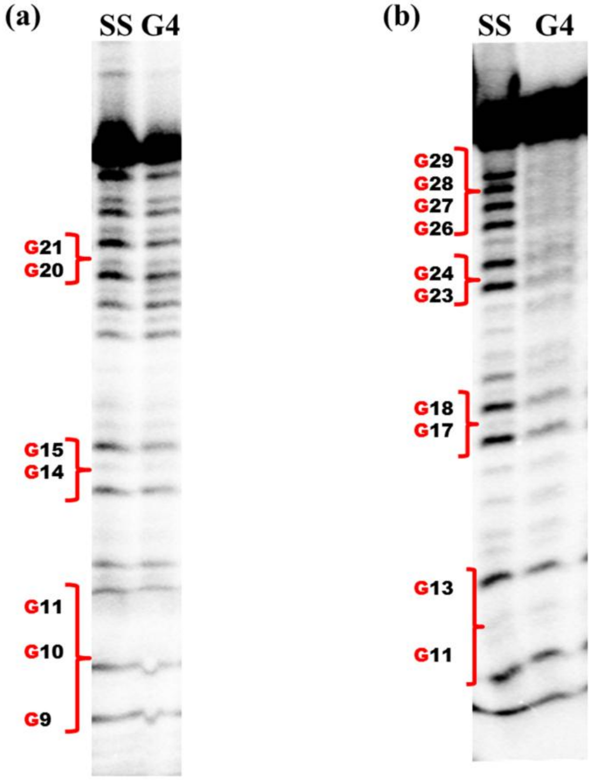

Supplementary Figure S14.1: ITC binding studies of TMPyP4 with HGQs-mutants. (a: HGQ-NV-Lmut; b: HGQ-NV-Gmut). The table below includes the thermodynamic parameters obtained due to interaction of the HGQ and their mutants with TMPyP4 with the help of ITC.

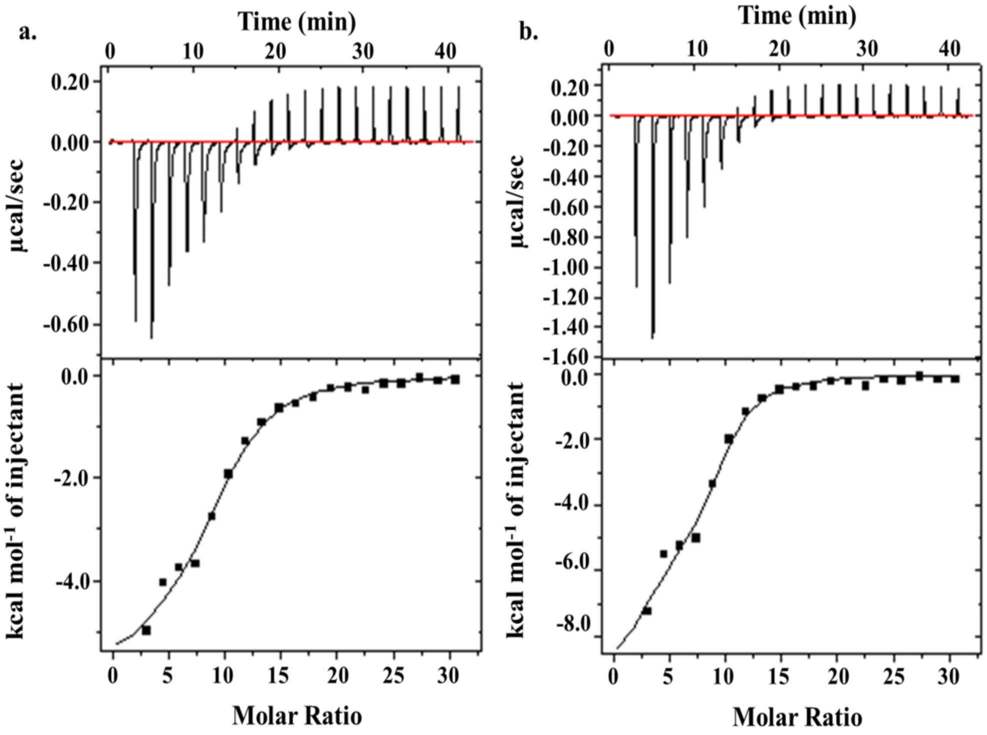

Parameters	HGQ-NV-L + TMPyP4	HGQ-NV-L-MUT + TMPyP4	HGQ-NV-G + TMPyP4	HGQ-NV-G-MUT + TMPyP4
**K_a1 (M^{-1})**	**1.0E8**	**1.49E5**	**3.32E8**	**6.31E5**
**K_a2 (M^{-1})**	**7.41E4**	**1.53E5**	**1.64E5**	**2.82E5**

Supplementary Figure S14.2: ITC binding studies of Braco-19 with HGQs and their corresponding mutants. (a: HGQ-NV-L; b: HGQ-NV-Lmut; c: HGQ-NV-G; d: HGQ-NV-Gmut). The table below includes the thermodynamic parameters obtained due to interaction of the HGQ and their mutants with Braco-19 with the help of ITC.

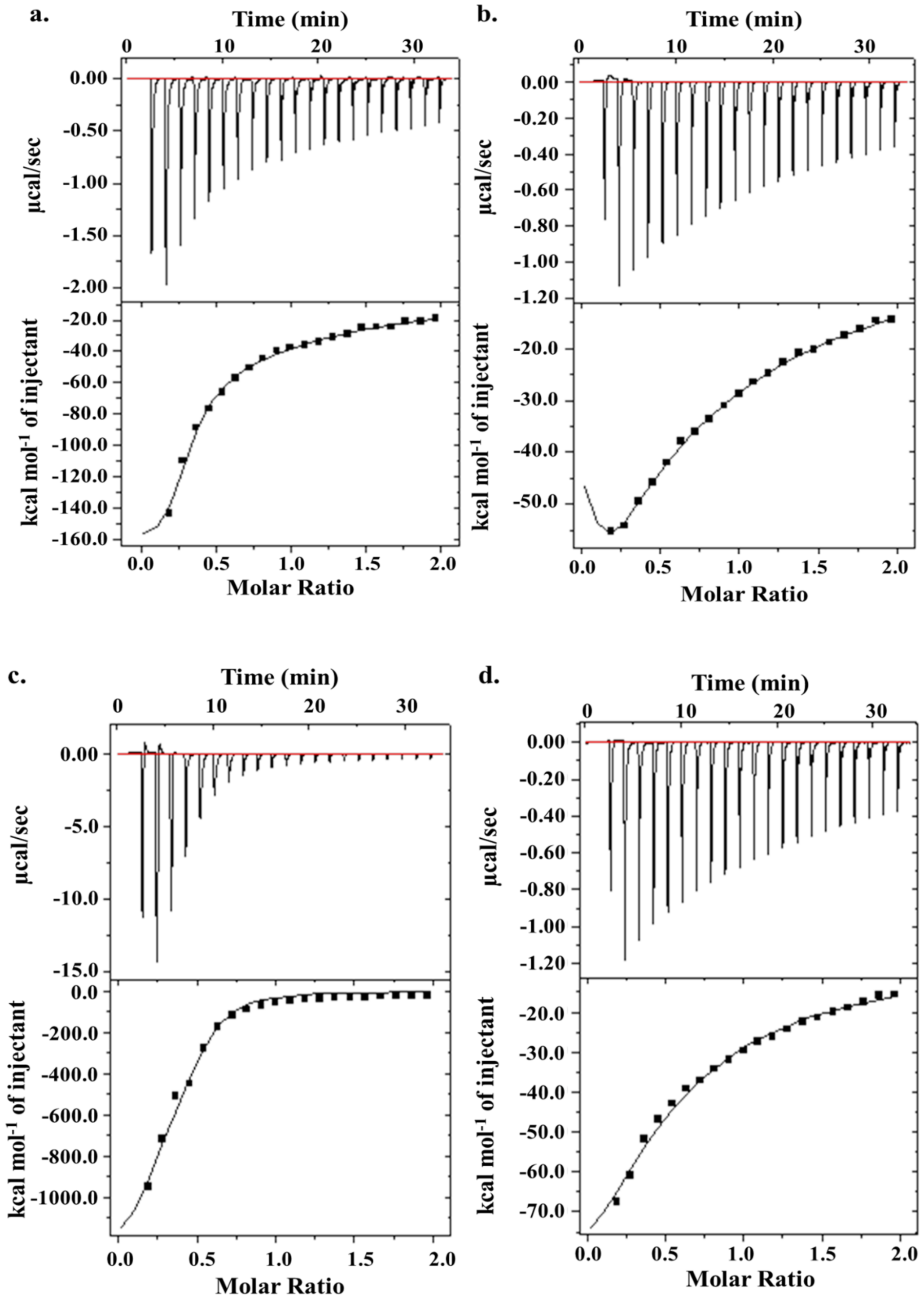

Parameters	HGQ-NV-L + Braco-19	HGQ-NV-L-MUT + Braco-19	HGQ-NV-G + Braco-19	HGQ-NV-G-MUT + Braco-19
K_a1 (M^{-1})	3.52E6	6.21E4	2.77E6	2.24E5
K_a2 (M^{-1})	4.58E4	1.35E6	2.73E6	4.45E4

Supplementary Figure S15: Taq DNA polymerase stop assay shows stalling due to the presence of a stable G-quadruplex. For the assay, the PRIMER-TPSA (Table S18) was ^{32}P-labelled. The template DNA i.e. NV-L-TPSA or NV-G-TPSA (10nM) was incubated with the labelled primer and cold primer (15 nM) in Tris-HCl buffer (100 mM KCl , pH 7.4) and heated at 95 °C for 5 min followed by slow cooling. The increasing amounts of Braco-19 was added to the reaction mixture and incubated for 30 min at 37°C. Finally, the polymerase reactions was initiated by adding the reaction buffer (5 mM $MgCl_2$, 1.5 mg/ml BSA, and 0.2 mM dNTPs) along with the Taq DNA polymerase (NEB) at 42°C for 1 hr.The reaction was topped by the addition of formamide loading dye and samples were heated at 95°C. The samples were analyzed in 15% urea denaturing PAGE. The gel were dried and exposed to the phosphorimaging screen overnight. The image was the obtained using the Fuji FLA-5000 phosphorImager.

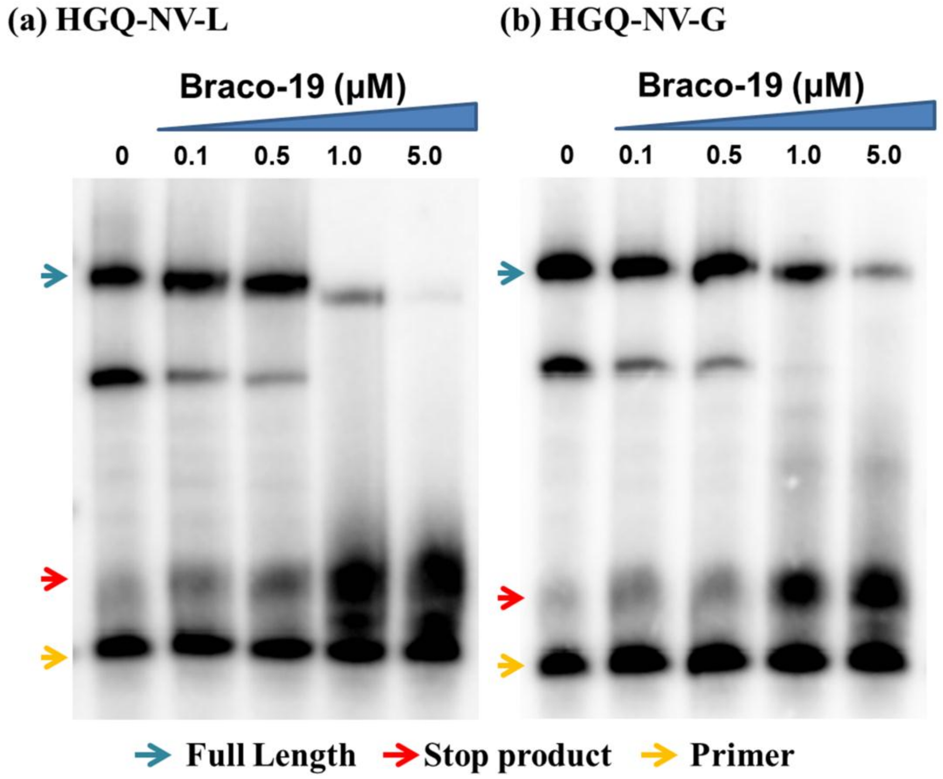

Supplementary Figure S16: CD melting plots obtained for the HGQs on the addition of Braco-19. (a) HGQ-NV-L with Braco-19 for D/N ratio=0, D/N ratio=1, and D/N ratio=2, (b) HGQ-NV-G with Braco-19 for D/N ratio=0, D/N ratio=1, and D/N ratio=2. The T_m difference between D/N ration =1 and D/N ratio =0 is represented as ΔT_m1 and the Tm difference between D/N ration =2 and D/N ratio =1 is represented as ΔTm2. Significant change in Tm was also observed for both the HGQs on addition of Braco-19.

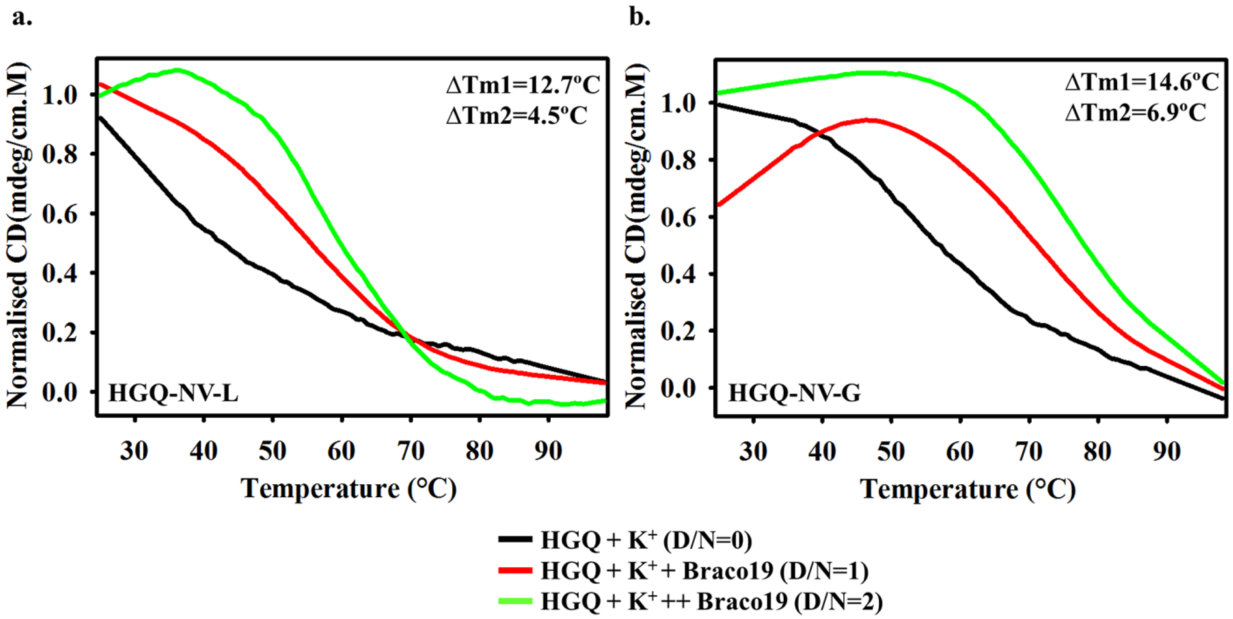

Supplementary Table S17: Dose-dependent effect of TMPyP4 on the expression of TFP bearing the HGQ sequence. Three different concentrations have been used 20 µM, 50 µM and 100 µM and we observed a gradual diminishment of fluorescence intensity with increase in ligand concentration. Thus, implicating the stabilization of the G-quadruplex sequence by the G-quadruplex binding ligand, TMPyP4.

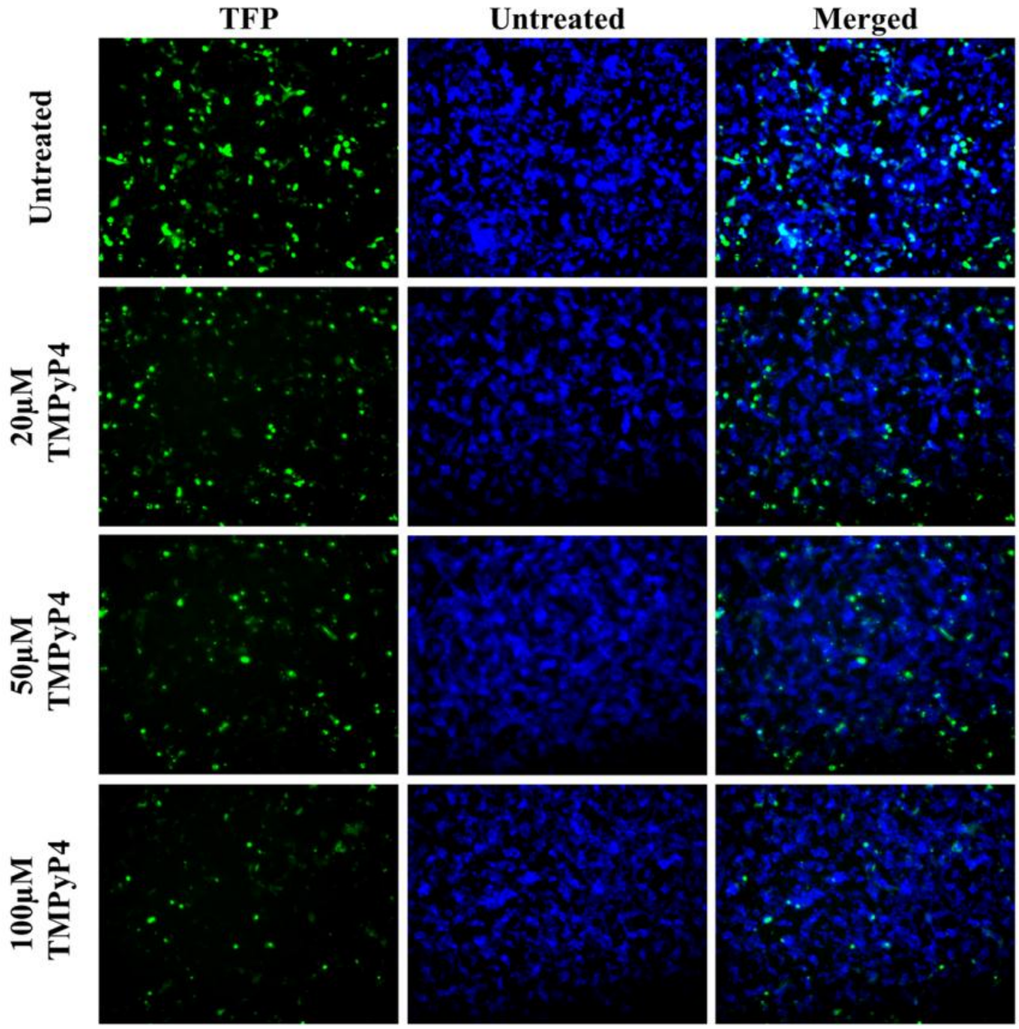

Supplementary Table S18: List of primers, templates and other oligonucleotides used in the experiments.

Sl. No.	Sequence Name	Sequence(5'----3')
1.	HGQ-NV-L	TTTAAGGAGAACGGGATGGTTAAGGATGAG
2.	HGQ-NV-Lmut	TTTAAGAAGAACGAAATTGTTAAAGATGAG
3.	HGQ-NV-G	ACAGTGGTAAACGGTGTTTGGATTTGGTGGGG
4.	HGQ-NV-Gmut	ACAGTAATAAACAGTGTTTCTATTTCGTGAGA
5.	HGQ-NV-L-F	ATGAGTGAATTCATGAGGAGAACGGGATGGTTAAGGG TGAGCAAGG
6.	HGQ-NV-G-F	ATGAGTGAATTCATGGTGGTAAACGGTGTTTGGATTTG GTGAGCAAGG
7.	HGQ-NV-Lmut-F	ATGAGTGAATTCATGAGAAGAACGAAATTGTTAAAGG TGAGCAAGG
8.	HGQ-NV-Gmut-F	ATGAGTGAATTCATGATAATAAACGATGTTTCTATTTC GGTGAGCAAGG
9..	HGQ-NV-RP	CGTGCTAGCCTTGTACAGCTCGTCCATGCC
10.	HGQ-NV-PSA-FP	ATCCATGAGTGAATTCATG
11.	HGQ-NV-PSA-RP	TTCTAATCCTTGCTCACC
12..	HGQ-NV-mut-PSA-RP	TTCTAATCCTTGCTCACTT
13.	Bcl2	AGGGGCGGGCGCGGGAGGAAGGGGGCGGGAGCGGGG CTG
14	c-Myc	TGAGGGTGGTGAGGGTGGGGAAGG
15	NV-L-TPSA	AATATTTTAAGGAGAACGGGATGGTTAAGGATGAGCA CGACACGCAATTGCTATAGTGAGTCGTATTA
16	NV-G-TPSA	GCACTACAGTGGTAAACGGTGTTTGGATTTGGTGGGGT CCAGCACGCAATTGCTATAGTGAGTCGTATTA
17	PRIMER-TPSA	TAATACGACTCACTATAGCAATTGCGTG
18	HGQ-NV-L-DMS	AAGGAGAACGGGATGGTTAAGGAT
19	HGQ-NV-G-DMS	GTGGTAAACGGTGTTTGGATTTGGTGGGGTC

Supplementary Figure S19: Full length gel images of Figure 3b showing electrophoretic gel mobility shift assay.

Lane 3 and 8 denote the mobility of the mutant counterparts of the G-quadruplex, Lane 4, 5, 6 and 7 denote the mobility of HGQs in the presence of K^+, Na^+, Li^+ and Mg^{2+}respectively. Lane 2 represents the mobility of G-quadruplex forming sequence (positive controls): c-Myc or Bcl2. Lane 1, negative control of c-Myc or Bcl2, i.e., the mutant sequence of c-Myc or Bcl2 gene (a: HGQ-NV-L and b: HGQ-NV-G).

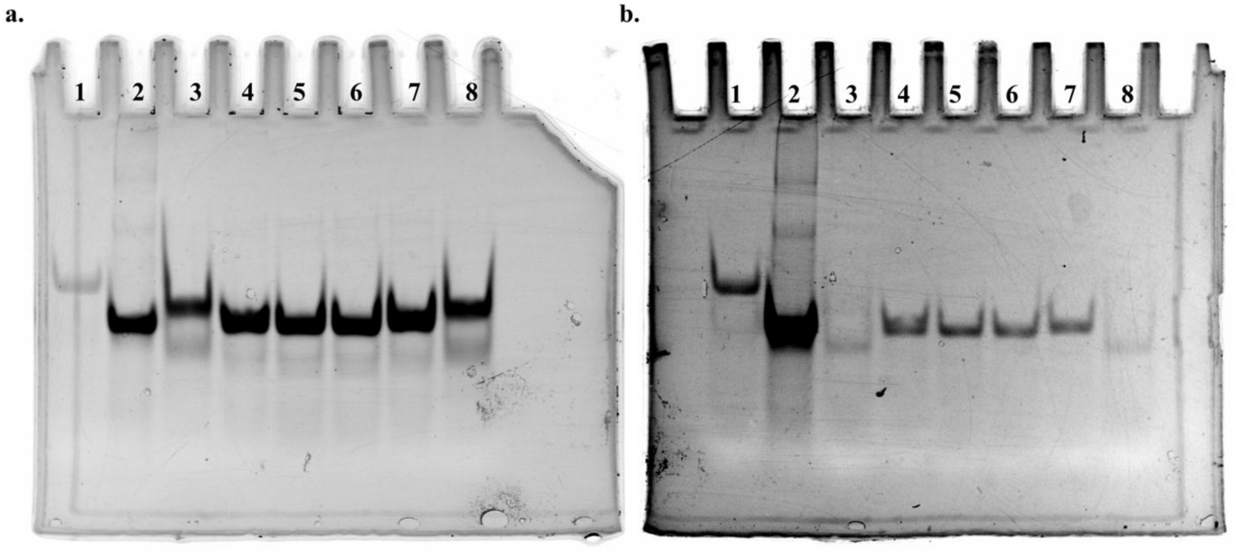

Supplementary Figure S20a-b: Full length gel images of Figure 6 showing **Primer extension assay.** This assay shows a decrease in intensity of the band with increasing concentration of TMPyP4 for both the HGQs (a1: HGQ-NV-L with TMPyP4; a2: HGQ-NV-L with TMPyP2; a3: HGQ-NV-Lmut with TMPyP4; b1: HGQ-NV-G with TMPyP4; b2: HGQ-NV-G with TMPyP2 and b3: HGQ-NV-Gmut with TMPyP4)

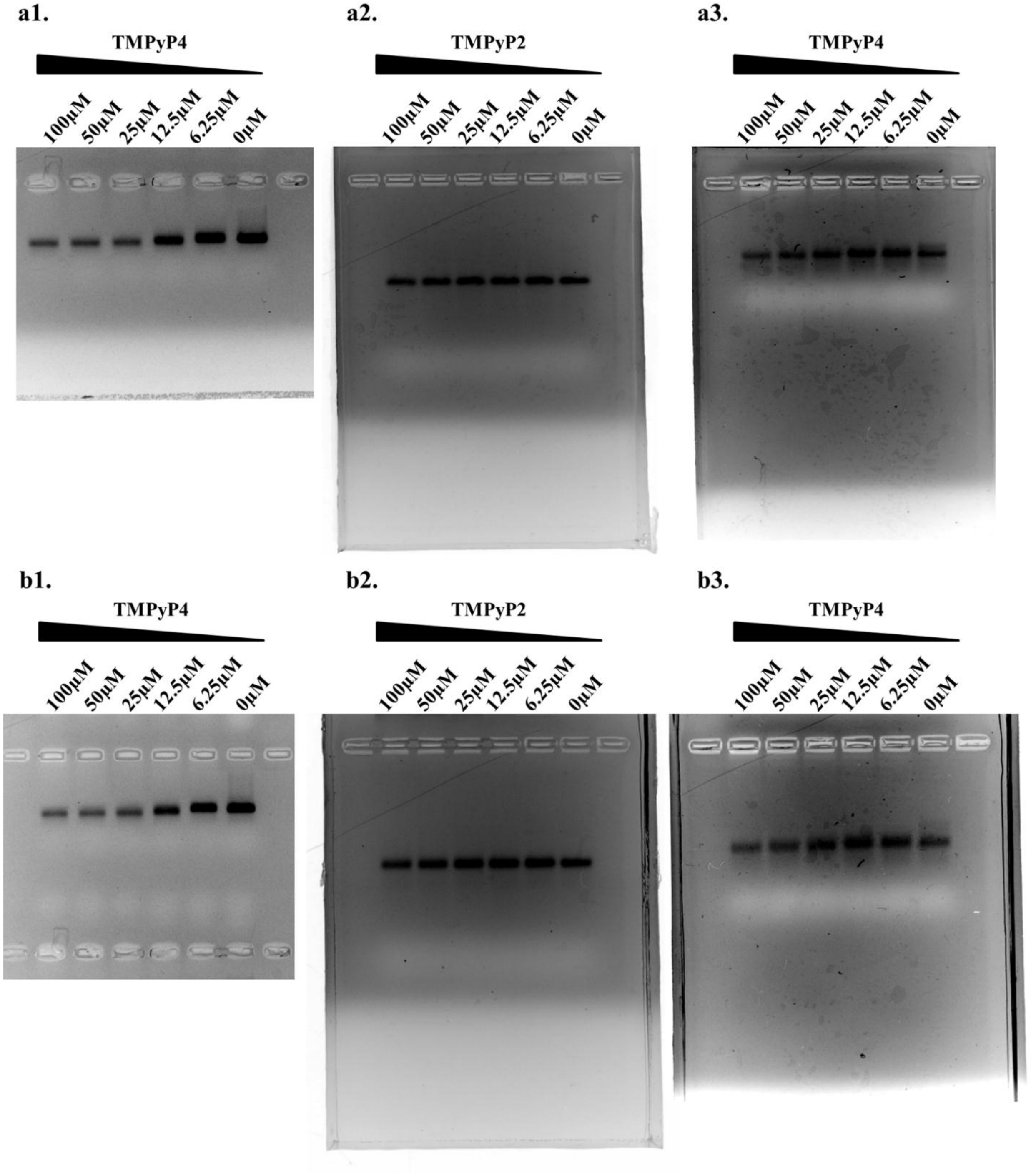

Supplement: Supplementary file 1 — Supplementary Information. [file 41598_2020_58406_MOESM1_ESM.pdf]
